# Modelling learning dynamics in autism therapy through explainable multimodal representation learning

**DOI:** 10.3389/fnins.2026.1737098

**Published:** 2026-07-14

**Authors:** Patrick O. Akinwumi, Meihua Qian, Stephen Ojo

**Affiliations:** 1 College of Education, Clemson University, Clemson, SC, United States; 2 College of Engineering Anderson University, Anderson, SC, United States

**Keywords:** autism spectrum disorder, behavioural primitive, multimodal behaviour modeling, pose and optical flow, therapy session analysis, unsupervised representation learning

## Abstract

**Background:**

Autism Spectrum Disorder (ASD) presents with complex, temporally evolving motor and social behaviours that are difficult to quantify in ecologically valid clinical contexts. While recent computational methods offer diagnostic insights, many depend on fully supervised learning, high-resolution video, or artificial experimental constraints, limiting scalability, interpretability, and privacy compliance. Few approaches leverage unsupervised models to uncover dynamic behavioural structure from minimally invasive inputs.

**Methods:**

To address these limitations, we propose a privacy-preserving, unsupervised representation learning framework that operates solely on skeletal pose and optical flow features. Using 255 multimodal windows from 15 therapy sessions in the MMASD corpus, a publicly available, privacy-safe dataset of child-clinician interactions, we train a denoising temporal autoencoder to derive compact latent embeddings of behaviour.

**Results:**

The model uncovers a low-dimensional behavioural manifold composed of six latent motor clusters. Transition graphs reveal structured topologies, including behavioural hubs and bottlenecks. Saliency analyses identify anatomically and socially relevant features, such as joint pairs (LWrist-LAnkle, Neck-Rear Head) and dynamic flow regions (e.g., index pair 7, 14). Temporal saliency, based on reconstruction error, highlights spontaneous gesture onsets and socially salient events. KL divergence between early and late session phases quantified intra-session adaptation (range: 0.06–17.7) and showed a strong negative correlation with joint attention duration (r = −0.96, *p* = 0.002), suggesting links between behavioural dynamics and social engagement.

**Discussion:**

These findings offer preliminary evidence that interpretable behavioural structure can be extracted from low-resolution, privacy-compliant inputs. While based on a limited sample, the framework illustrates potential for modeling learning dynamics, identifying salient motor patterns, and supporting objective progress tracking in ASD therapy. Future work will involve clinical validation and application to larger, longitudinal datasets to assess generalizability and therapeutic utility.

## 1 Introduction

Autism Spectrum Disorder (ASD) is a multifaceted neurodevelopmental condition characterized by persistent deficits in social communication and interaction, alongside restricted and repetitive behaviours, interests, or activities, as defined by the Diagnostic and Statistical Manual of Mental Disorders (DSM-5) (Hassan et al., 2025; Rodgers and McCluney, 2021; Visser et al., 2016; Volkmar and McPartland, 2014). Symptoms manifest in early development and cause significant impairments in social, occupational, or other functional domains (Lord et al., 2000). The DSM-5 identifies three levels of support needs: Level 1 (requiring support), Level 2 (substantial support), and Level 3 (very substantial support) (Gardner et al., 2018; Volkmar and McPartland, 2014). Although the precise etiology of ASD remains unclear (Shaw et al., 2014), it is generally attributed to complex interactions between genetic and environmental factors (Cheroni et al., 2020; Sealey et al., 2016). Genetic variations influencing neurodevelopment, as well as prenatal and perinatal environmental exposures, contribute to structural and functional brain differences in individuals with ASD (Bai et al., 2019; Bölte et al., 2019). Globally, ASD affects approximately one in 68 children, representing a significant public health concern (Hossain et al., 2017). Epidemiological studies suggest a worldwide increase in ASD prevalence over the past fifty years, rising from 6.7 to 23.0 per 1,000 children from 2000 to 2018 (Maenner, 2023). Due to the complex and varied nature of ASD, diagnosis requires a multi-dimensional approach (Abdollahinejad and Kabir, 2024; Lai et al., 2015).

The disorder imposes considerable social, emotional, and financial burdens on affected families and healthcare systems, and an estimated 317 million children and adolescents were living with developmental disabilities in 2019, with the highest burdens found in low- and middle income countries (LMICs) (Hus and Segal, 2021; Stewart and Lee, 2017). These conditions disrupt various domains of development-including cognition, language, motor skills, and social interaction, and often require lifelong support and intervention. Early diagnosis and intervention are thus critical to improving developmental outcomes and overall quality of life (Lai et al., 2015), as timely interventions have been shown to markedly improve outcomes in language acquisition, adaptive behaviour, and academic success (Scherzer et al., 2012).

ASD is typically diagnosed between the ages of two and four, when developmental delays in communication, socialization, and behaviour become more apparent (Landa et al., 2007). Early indicators, such as reduced eye contact, limited verbal skills, and repetitive movements, often prompt clinical evaluation (Yoo, 2016). Individuals with ASD face persistent challenges that impede social relationships, academic progress, and employment opportunities, ultimately affecting their quality of life (Shaw et al., 2014). Comorbidities such as anxiety, depression, and epilepsy occur in up to 70% of cases, further complicating management (Al-Beltagi, 2021). Without timely and appropriate support, these challenges may lead to isolation, mental health difficulties, and decreased autonomy (King et al., 2020). While interventions such as Applied Behaviour Analysis (ABA), speech-language therapy, occupational therapy, and Individualized Education Programs (IEPs) are widely implemented and have shown promise in early, multidisciplinary contexts (Chung et al., 2024; Sengupta et al., 2017), the broader empirical literature reports mixed and often modest effects. However, recent meta-analyses suggest that outcome variability and methodological heterogeneity warrant cautious interpretation (Bottema-Beutel et al., 2023; Steinbrenner et al., 2020; Bottema-Beutel et al., 2019; Sandbank et al., 2020).

Despite the potential for early identification, as young as 14 months (Barbaro and Dissanayake, 2009), over 70% of children receive diagnoses after 51 months (Barbaro and Dissanayake, 2009). Early intervention is directly associated with improved cognitive outcomes, with children diagnosed before age two showing IQ gains of up to 20 points within the first year of therapy (Khan and Katarya, 2025; Schmidhuber, 2015), whereas later diagnoses yield minimal benefit (Robain et al., 2020). The Autism Diagnostic Observation Schedule (ADOS) remains the “gold standard” for diagnosis (Lord et al., 2000), but its reliance on clinician expertise introduces subjectivity and inconsistency (De Belen et al., 2020; Taylor et al., 2017). Nevertheless, ADOS exhibits reduced validity for nonspeaking or minimally verbal individuals, particularly those with motor-speech impairments, highlighting the need for alternative behavioural frameworks (Bottema-Beutel et al., 2019).

Limited access to trained professionals contributes to prolonged diagnostic delays, averaging 48 months for initial assessment and 61 months for final diagnosis (Wiggins et al., 2006). These constraints underscore the need for objective, scalable diagnostic tools that can operate independently of clinician bias.

Traditional behavioural assessments, while essential, inadequately capture ASD’s complex heterogeneity and overlapping symptomatology with other neurodevelopmental disorders (NDDs) such as ADHD (Egbuna et al., 2025; Gillberg, 2021; Visser et al., 2016). Machine learning (ML) and computer vision (CV) techniques have emerged as promising alternatives for identifying behavioural and neurobiological markers of ASD, as traditional clinical assessment such as ADOS and the Autism Diagnostic Interview-Revised (ADI-R) are costly, time-consuming, and subjective (De Belen et al., 2020; Schmidhuber, 2015). CV enables the automated analysis of nonverbal cues, gait, posture, gestures, facial expressions, and eye gaze, offering potential digital biomarkers for early detection and therapy evaluation (De Belen et al., 2020). For instance, Ganai et al. (2025) demonstrated that gait analysis through CV can serve as a non-invasive motor biomarker for early ASD identification, reducing reliance on costly motion-capture systems. Similarly, De Belen et al. (2020) reviewed CV applications in ASD and found that vision-based methods objectively quantify behavioural and biological markers, enhancing diagnostic reliability. These advancements extend beyond diagnosis to therapeutic contexts, supporting adaptive interventions and social skills training.

The integration of multimodal data, including behavioural, neuroimaging, and physiological inputs, can further improve diagnostic precision and personalize therapeutic approaches (Gkintoni et al., 2025). However, the opacity of many AI models limits their clinical adoption. Explainable Artificial Intelligence (XAI) bridges this gap by combining computational efficiency with interpretability, fostering clinician trust in data-driven decision systems. This study thus aims to model learning dynamics in ASD therapy through explainable multimodal representation learning, addressing current challenges in diagnostic accuracy, interpretability, and clinical applicability. This approach draws on foundational work in representation learning that demonstrates the value of unsupervised embeddings for capturing structured patterns in complex behavioural data (Bengio et al., 2013; Zhao et al., 2020).

## 2 Literature review

Diagnosing Autism Spectrum Disorder remains a major challenge due to its clinical heterogeneity and the inherent subjectivity of traditional behavioural assessments. Although these assessments are valuable, they often fail to capture the full range of ASD manifestations, particularly in atypical presentations resulting in diagnostic delays and reduced intervention efficacy (Cruz Puerto and Sandín Vázquez, 2024; Lai et al., 2015). To address these limitations, recent research has explored the integration of biological data such as genetic, neuroimaging, and phenotypic measures (Bellosta and Soldano, 2019; Ecker and Murphy, 2014). However, single-modality analyses lack the comprehensiveness needed to characterize ASD’s multidimensional nature, underscoring the importance of multimodal diagnostic frameworks (Lai et al., 2015; Shamhan et al., 2025).

Neuroimaging techniques, including anatomical MRI (aMRI), functional MRI (fMRI), and diffusion tensor imaging (DTI), have provided critical insights into the neurobiological basis of ASD by revealing atypical brain structure and connectivity patterns. Nevertheless, variability in imaging protocols, small sample sizes, and inconsistent preprocessing limit their generalizability and clinical application. Phenotypic data offer complementary behavioural and developmental context but insufficient depth to elucidate underlying neural mechanisms (Abdollahinejad and Kabir, 2024). Integrating these data sources within a multidimensional learning framework has been shown to enhance diagnostic precision and reliability (Dcouto and Pradeepkandhasamy, 2024).

Artificial Intelligence (AI), particularly machine learning (ML) and deep learning (DL) methods, has gained prominence in advancing multimodal ASD diagnostics. These models can uncover latent patterns across heterogeneous datasets, improving predictive accuracy and facilitating individualized characterization. Explainability methods, such as Shapley Additive Explanations (SHAP), further ensure clinical interpretability by identifying which features most influence diagnostic decisions (Miao et al., 2024). Building on this foundation, the present study develops an explainable, multimodal pipeline for modelling behavioural dynamics in autism therapy.

Functional MRI (fMRI) has been particularly valuable for examining aberrant neural connectivity and functional organization in ASD. Resting-state fMRI (rs-fMRI), which captures spontaneous neural activity, provides rich data for identifying disrupted brain networks characteristic of neurodevelopmental disorders (NDDs) (Khosla et al., 2019b). However, rs-fMRI data are inherently high-dimensional and noisy, requiring advanced AI methods for effective feature extraction and pattern recognition. Khosla et al. (2019a) for instance, employed a graph-based deep learning approach to model inter-regional brain connectivity, achieving over 75% classification accuracy in distinguishing ASD from typically developing controls. Similarly, attention-based architectures have been introduced to dynamically identify the most discriminative neural features. Ahmadi et al. (2021) integrated temporal attention mechanisms to capture time-varying changes in connectivity, significantly improving ADHD classification performance. This shift from static to dynamic brain network modelling represents a critical evolution in computational neuropsychiatry, enabling the detection of transient yet clinically relevant disruptions in functional coordination that may underlie early developmental pathology.

### 2.1 Pose and movement analysis

Computer vision and pose-estimation methods have become valuable tools for quantifying motor and postural differences in individuals with ASD. Algorithms such as OpenPose, Mask R-CNN, and Kinect-based tracking extract two- or three-dimensional skeletal key points from video, enabling objective analysis of movement dynamics.

Dawson et al. (2018) used video-based CV to measure toddlers’ head motion during a “response-to-name” task, finding greater spontaneous head movement in ASD children. Similarly, Martin et al. (2018) observed atypical lateral head movements, particularly in yaw and roll, among ASD participants. Extending beyond head pose, Kojovic et al. (2021) trained an LSTM model on sequences of 2D skeletal key points from child-adult play interactions, achieving approximately 81% classification accuracy between ASD and typically developing groups. The use of de-identified skeletal data demonstrated that abstracted pose information can still capture diagnostic motor signatures.

Gait analysis studies, such as Ganai et al. (2025), have further quantified ASD-related motor deviations, reduced stride length, abnormal posture, and slower gait, using CV-based joint tracking. In therapeutic contexts, such quantitative movement features can assess engagement and motor progress during interventions. Pose-based CV provides a scalable, non-invasive means to capture gestural, postural, and locomotor markers associated with the social and sensorimotor characteristics of ASD (De Belen et al., 2020; Kojovic et al., 2021). This is particularly relevant for minimally verbal children, where motor-based indicators may provide more accessible and reliable signals of engagement and social intent than traditional language-based assessments (Bhat et al., 2011; McCleery et al., 2013; Yang et al., 2025).

### 2.2 Eye gaze and social visual behaviour

Eye gaze and facial attention provide critical insights into the social communication deficits characteristic of ASD. Individuals with ASD often exhibit atypical gaze patterns, including reduced eye contact and impairments in joint attention. Computer vision (CV)-based eye-tracking systems have been employed for both diagnostic assessment and therapeutic monitoring.

As reviewed by De Belen et al. (2020), children with ASD display distinct gaze behaviours, such as longer latencies to disengage and reduced fixation on faces, compared to neurotypical peers. Machine learning models leveraging these gaze features have successfully differentiated ASD from typical development by analysing viewing preferences for social versus non-social stimuli (Ahmed et al., 2023; Wei et al., 2024). While early systems primarily operated in controlled environments (e.g., children observing videos on screens), recent advances have focused on naturalistic interactions. Chong et al. (2017), for instance, developed a deep learning model, Pose-Implicit CNN, to detect eye contact during real adult-child play sessions using head-mounted cameras, achieving approximately 0.76 precision and 0.80 recall.

Extending these findings, Guo et al. (2023) proposed a social visual behaviour analytics framework for therapy contexts. Their model automatically detected mutual gaze, instances where both child and therapist-maintained eye contact, from play-based intervention videos. In a dataset of 28 children (84 clips, ~21 h), the model’s mutual gaze ratio closely matched human annotations, and predicted values correlated strongly with social engagement scores (Guo et al., 2023). These studies demonstrate the potential of vision-based algorithms to objectively quantify social visual behaviours, such as gaze direction, fixation, and facial expressivity, central to ASD diagnosis and therapy. Integrating gaze cues with complementary modalities (e.g., body orientation, gestures) enables a more comprehensive understanding of social engagement and learning dynamics in ASD.

### 2.3 Interactive and robotic interventions

Advancements in machine learning have facilitated the development of interactive therapy tools for ASD, particularly those using computer vision to monitor movements and deliver real-time feedback. Several studies have demonstrated the effectiveness of CV-enabled games and sensor-based systems in promoting emotional understanding, social engagement, and motor coordination among children with ASD. Harrold et al. (2014) designed emotion-recognition games in which on-screen avatars mimic or respond to a child’s facial expressions detected through CV, enhancing emotion identification skills. White et al. (2018) further reported strong engagement and user satisfaction among children participating in such emotion-training games.

Expanding to full-body interaction, Piana et al. (2019) employed an RGB-D camera to capture gesture-based movements during play, showing improved accuracy in emotion recognition with continued training. Similarly, Ringland et al. (2014) developed Sensory Paint, where children’s body movements controlled digital artwork, fostering attentional focus through embodied play. Magrini et al. (2015) introduced an audiovisual game translating arm movements into sounds, positively evaluated by both parents and therapists for its sensory feedback benefits (Magrini et al.). These CV-based, wearable-free systems demonstrate the therapeutic potential of gamified, interactive platforms for improving social and motor skills in ASD interventions.

In parallel, socially assistive robotics has emerged as a promising modality for adaptive ASD therapy. Robots equipped with vision systems can interpret children’s facial expressions, gaze, and gestures to provide responsive, context-aware interactions. Dickstein-Fischer and Fischer (2014) developed PABI (Penguin for Autism Behavioural Intervention), a vision-equipped robot designed to support joint attention and imitation training through visual tracking of facial and gestural cues. Esubalew et al. (2012) built a humanoid robot capable of interactive play sessions, using vision to guide engagement with ASD children. Mazzei et al. (2012) employed social robot-based treatment for subjects, outfitted with head-mounted cameras, to analyse multimodal behavioural responses during therapy. These systems adapt in real time to social signals such as gaze direction or smiles, enhancing therapeutic responsiveness.

As noted by De Belen et al. (2020), socially assistive robots provide a unique advantage by delivering multimodal cues, eye contact, facial expression, and imitation, within a single, integrated framework. The combination of computer vision and robotics thus offers a clinically relevant pathway for personalized, engaging, and scalable ASD interventions.

### 2.4 Unsupervised learning and behavioural phenotyping

Machine learning (ML) has become a central tool in advancing ASD diagnosis and intervention, with studies exploring diverse modalities, algorithms, and data sources. While supervised learning approaches have dominated autism spectrum disorder research, particularly for diagnostic classification, unsupervised learning is increasingly recognized for its ability to uncover latent structures in unlabelled data. Unsupervised representation learning enables models to automatically extract compact, informative features from high-dimensional behavioural data without requiring manual labels, making it particularly well-suited for analysing complex social interactions in clinical settings (Bengio et al., 2013; LeCun et al., 2015). Temporal autoencoders, in particular, have shown promise in capturing dynamic structure across time, allowing latent embeddings to reflect salient spatiotemporal patterns relevant to domains such as affective computing and developmental disorder assessment (Hinton and Salakhutdinov, 2006; Roth et al., 2016). Techniques such as clustering, dimensionality reduction, and latent embedding are particularly valuable for ASD, given the disorder’s pronounced heterogeneity. Parlett-Pelleriti et al. (2023) reviewed applications of unsupervised machine learning in ASD, emphasizing that vast datasets, such as therapy logs and sensor streams, often lack predefined labels yet contain rich latent information that can reveal meaningful subtypes and behavioural trends.

Stevens et al. (2019) applied Gaussian mixture and hierarchical clustering to a cohort of 2,400 children with ASD, identifying 16 behavioural subgroups and two overarching phenotypes with distinct symptom profiles. Notably, these data-driven clusters corresponded to differential treatment responses, and incorporating cluster membership improved regression models predicting skill gains, suggesting the utility of clustering for personalized intervention. Similarly, Gardner-Hoag et al. (2021) used k-means clustering on behavioural records from 854 children, identifying seven subgroups dominated by specific behaviours such as aggression or self-injury. Children in self-injury/aggression clusters demonstrated poorer treatment outcomes, indicating that such unsupervised phenotyping could guide more targeted interventions. These studies underscore the potential of unsupervised ML to delineate clinically meaningful ASD subtypes and inform precision therapy strategies.

Most existing applications analyse retrospective clinical or questionnaire-based datasets, whereas direct unsupervised analysis of sensor or video data remains limited. The primary challenge lies in the high dimensionality of vision-derived features. Autoencoder-based architectures address this issue by learning compact latent representations. In related neuroscience work, convolutional autoencoders have effectively compressed fMRI and EEG data for ASD-related analyses (Parlett-Pelleriti et al., 2023). Extending this to behavioural research, autoencoders could be applied to pose sequences or multimodal features to discover recurrent movement trajectories reflective of engagement or stereotypy. Although no studies to date have applied such models directly to video-based therapy data, advances in self-supervised learning, such as Transformer models with masked prediction, highlight the potential of these approaches (Leo et al., 2019). Unsupervised and self-supervised models hold promise for identifying latent digital phenotypes, subtle, emergent patterns of gaze, gesture, or interaction, that may correspond to engagement levels or therapeutic progress, offering a scalable path toward individualized ASD assessment and intervention.

### 2.5 Related empirical work

Early works (Zhao et al., 2020) introduced complex network measures as diagnostic features within ML frameworks, achieving 70.1% accuracy and demonstrating the potential of network-based neurobiological modelling for ASD prediction. Complementary research (Eldin Rashed et al., 2025) provided a comprehensive review of ML applications in ASD, comparing datasets of varying demographic and clinical characteristics and evaluating feature selection and classification methods. Subsequent investigations underscored the effectiveness of deep learning models: (Weng et al., 2017) reported that convolutional neural networks (CNNs) outperformed traditional classifiers such as support vector machines (SVMs) and random forests, reaching accuracies of 99.53% for adults, 98.30% for children, and 96.88% for adolescents. Similarly, Twala and Molloy (2023) demonstrated that ensemble methods, particularly bagging, enhanced prediction reliability in robot-assisted therapy contexts, reducing error rates to 21.4%.

Neuroimaging-based studies have integrated graph theory and ML to characterize ASD using multiparametric MRI datasets (Twala and Molloy, 2023). These models capture structural and functional connectivity patterns, achieving high accuracy in identifying ASD-specific neurobiological signatures. Advances in explainable and privacy-preserving AI have further strengthened interpretability and data security. Vidya et al. (2024) proposed an explainable AI pipeline that identified key fMRI biomarkers aligned with clinical knowledge, while GAMI-Net (Malik et al., 2025) introduced interpretable probabilistic behavioural embeddings bridging automated predictions and clinical reasoning. Federated learning (FL) frameworks have also emerged: Shamseddine et al. (2022) developed privacy-preserving FL models that achieved 70% accuracy using behavioural traits and 62% using facial features, and Farooq et al. (2023) reported 98% accuracy for children and 81% for adults through meta-classifier aggregation, underscoring FL’s value for secure, distributed ASD detection.

The availability of large-scale neuroimaging datasets, such as the Autism Brain Imaging Data Exchange (ABIDE) (Di Martino et al., 2014), has facilitated reproducible cross-site evaluations. Recent works have evolved from unimodal pipelines [e.g., ASD-DiagNet (Daniel et al., 2025; Giansanti, 2023); DVAE (Pagnozzi et al., 2018)] to multimodal fusion frameworks integrating fMRI, sMRI, and behavioural data. Sophisticated models such as Gao and Song’s HE-MF, combined attention-based multimodal fusion with hierarchical connectivity features, achieving over 95% accuracy. Transformer-based architectures, including MCBERT (Khan and Katarya, 2025), further enhanced cross-site generalization, reaching 93.4% accuracy in ABIDE evaluations. Researchers have emphasized that structured multimodal fusion, whether at early, intermediate, or late stages, outperforms naïve feature concatenation. Graph-based frameworks such as dual-transformer and multi-view graph convolutional networks (GCNs) (Song et al., 2024) have also demonstrated strong performance (AUC = 0.85), effectively modelling spatial–temporal connectivity and subject heterogeneity.

Emerging trends include the use of generative adversarial networks (GANs), reinforcement learning, and hybrid architectures for robust multimodal integration (Ali et al., 2025; Usmani et al., 2024). Vision-based CNN frameworks such as GM-VGG-Net have achieved high gray-matter classification accuracy, although limited by the absence of behavioural integration. Surveys and umbrella reviews (Malik et al., 2025) consistently highlight two persistent gaps: (i) insufficient explainability and personalization, and (ii) limited clinical validation.

Prior work has leveraged skeletal motion features to identify characteristic motor patterns in ASD (Kindregan et al., 2015; Leekam et al., 2007). For instance, Saha et al. (2021) extracted linear and angular features from skeletal data, while Wang and Yang (2022) applied attention-based learning to 3D skeletons for engagement classification. Wang et al. (2025) provide a comprehensive review of visual human behaviour sensing techniques for ASD treatment, highlighting advances in gesture tracking, facial dynamics, and pose-based interaction modeling. Our current study focuses solely on unsupervised modelling of behavioural dynamics from skeletal pose and optical flow data, without attempting clinical prediction tasks such as ASD severity or ADOS scoring. Future extensions may explore multimodal fusion and clinical outcome prediction, but the present framework is limited to modelling behavioural structure and within-session dynamics.

Beyond ASD-specific applications, related advances in unsupervised behavioural representation learning across other domains further support our approach.

Beyond ASD, unsupervised behavioural representation learning has advanced in fields such as human activity recognition, video understanding, and social robotics. Techniques including temporal contrastive learning, motion transformer architectures, and graph-based skeleton modelling have shown promise in modelling interaction dynamics and latent behavioural structure (Arnab et al., 2021; Liu et al., 2025; Qian et al., 2021; Toaiari, 2025; Yan et al., 2018; Yang et al., 2026; Zhou et al., 2018). These approaches inform the present study’s design and motivate the use of attention-based sequence modelling for real-world behaviour segmentation.

### 2.6 The present study

To address these gaps, the present study integrates unsupervised deep learning with pose and motion-based features to develop ecologically valid, interpretable models of ASD behaviour. Drawing on Parlett-Pelleriti et al. (2023), we employ unsupervised methods, such as pose autoencoders and trajectory clustering, to discover latent behavioural patterns in unlabelled video data. Our framework uses 8-frame temporal windows (stride = 4) to balance temporal resolution and model stability. Denoising is performed via Gaussian jitter and dropout, simulating real-world variability in movement and camera jitter. The framework operates solely on 2D skeletal pose and optical flow features, preserving privacy while capturing dynamic indicators of engagement and reciprocity. While prior studies have explored multimodal cues such as gaze, audio, and facial key points, these were not implemented in the current version and are a key direction for future work. To ensure interpretability and robustness, we assess the stability of saliency outputs across multiple seeds and normalization schemes. Our approach is trained on naturalistic therapy sessions (e.g., play-based interventions), enhancing robustness under real-world conditions. The resulting trajectory-based metrics provide interpretable summaries of within-session behavioural change. Future studies should validate these metrics against standardized clinical assessments such as ADOS scores to fully anchor computational insights to therapeutic outcomes.

## 3 Methods

### 3.1 Dataset and ethical rationale

This study utilizes the MMASD corpus (“A Multimodal Dataset for Autism Intervention Analysis”) (Li et al., 2023), a publicly available, privacy-preserving dataset curated to support computational analysis of autism therapy sessions. The dataset comprises non-identifiable behavioural modalities, including optical flow, 2D skeletal key points, 3D pose trajectories, and structured clinician annotations, thereby safeguarding participant anonymity while retaining clinically relevant behavioural information. By omitting raw facial or audio recordings, MMASD adheres to ethical best practices in autism research and aligns with increasing demands for privacy-aware AI applications in healthcare (Dos Santos Melicio et al., 2025).

Since the released version contains only teaser and sample sequences in GIF format, we programmatically enhanced temporal resolution by extracting frame-level data to recover motion continuity. Subsequent analyses focused exclusively on these de-identified data streams, flow and pose, ensuring that all modelling efforts remained within ethically bounded, non-identifiable inputs, in accordance with the dataset’s design intentions.

To interpret clusters, we denormalized representative windows and mapped their centroid embeddings to real-time video frames (pose overlays only), enabling visual inspection of recurrent behavioural motifs such as reaching, rocking, or leaning.

### 3.2 Preprocessing and feature extraction

#### 3.2.1 Frame extraction and optical flow

To recover temporal dynamics from the sample GIFs, individual frames were first decoded. Dense optical flow was then computed between successive frames using the Farnebäck algorithm as implemented in OpenCV. For each frame pair, motion was summarized using three statistical descriptors, mean, standard deviation, and median of flow magnitude, yielding a compact representation resilient to minor spatial noise.

Farnebäck’s polynomial expansion model remains a widely accepted approach for estimating two-frame motion under challenging visual conditions (Farnebäck, 2003) and has been effectively applied in low-resolution environments such as classroom and therapy footage. Its balance of computational efficiency and accuracy renders it particularly suitable for real-time or privacy-constrained behavioural analytics in autism intervention contexts.

#### 3.2.2 2D skeletal key points (privacy-preserving pose)

To preserve privacy while capturing essential behavioural structures, 2D body landmarks were extracted using MediaPipe BlazePose, which tracks 33 anatomical joints per frame. BlazePose is part of the MediaPipe framework, a robust, cross-platform computer vision library offering real-time, production-ready pose estimation optimized for human pose in constrained settings such as therapy environments (Bazarevsky et al., 2020).

**Figure 1:**
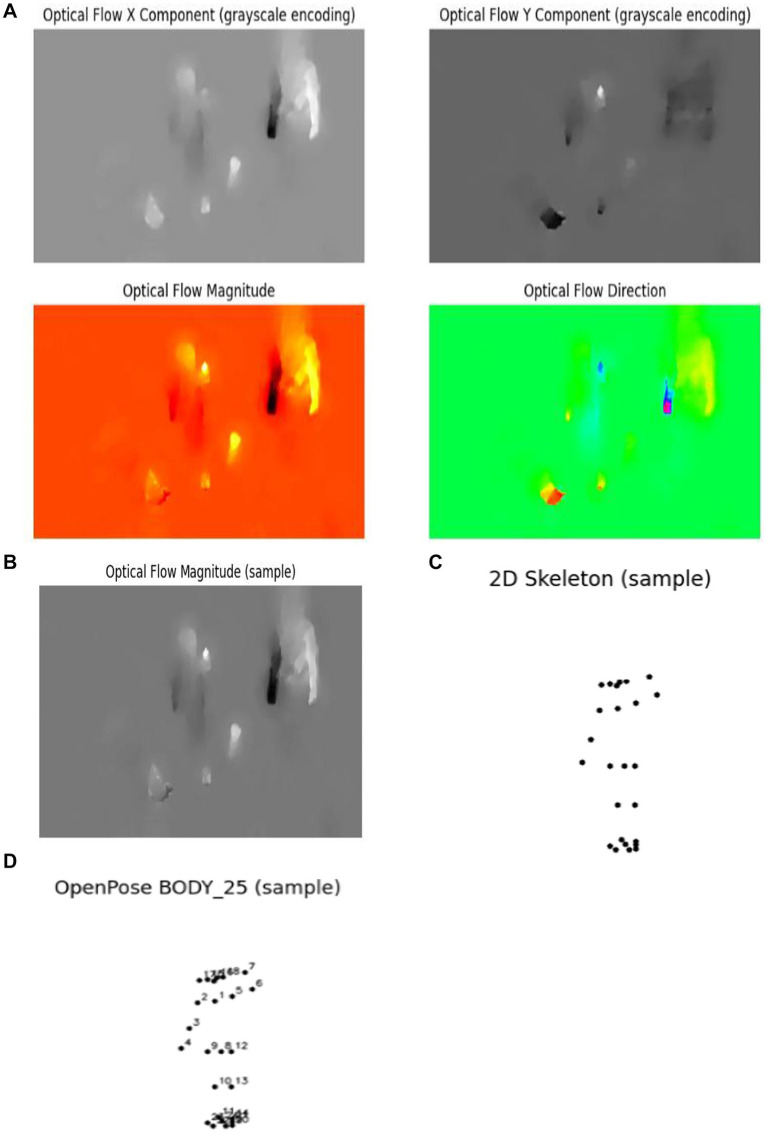
Privacy-preserving behavioural representations via optical flow and pose modalities. **(A)** Optical flow representations decompose motion into grayscale-encoded X and Y components, magnitude, and direction. These channels isolate dynamic patterns in the interaction space while abstracting personal identity. **(B)** Grayscale rendering of optical flow magnitude captures zones of high movement intensity, highlighting transient motion bursts. **(C)** Sample 2D skeletal pose with anonymized joint coordinates extracted using MediaPipe BlazePose. **(D)** OpenPose BODY_25 rendering enumerates anatomical keypoints used only for illustrative purposes. All analytic computations in the study used MediaPipe BlazePose (33 joints).

Rather than relying on raw (x, y, visibility) coordinates, we transformed the skeletal output into a privacy-preserving geometric signature. Specifically, we computed all pairwise Euclidean distances among joints and applied ℓ₂ normalization by the maximal distance per frame to ensure scale invariance. This approach discards individual appearance while preserving pose structure, aligning with prior work emphasizing geometry-based representations for ethical behaviour modelling. Note: While MediaPipe BlazePose (33 joints) was used exclusively for all model inputs and analysis, OpenPose BODY_25 (25 joints) was retained for select figure visualizations (e.g., Figure 1D) to enhance visual clarity. These renderings played no role in training or evaluation.

#### 3.2.3 Modality alignment and temporal windowing

Frame-level pose and optical flow features were temporally aligned by truncating to the shorter stream, then concatenated to form synchronized multimodal vectors. Sequences were segmented into overlapping windows of 8 frames (stride = 4), capturing short-range behaviours such as gestures and imitation. The 8-frame window (approximately 0.3 s at 30fps) balances temporal resolution with memory constraints and has been shown to effectively capture micro-behaviours in child movement. Stride = 4 introduces 50% overlap, improving temporal sensitivity while reducing redundancy. All features were z-scored to ensure stable optimization, standard in time-series learning.

#### 3.2.4 Representation learning

We employed a denoising temporal Transformer autoencoder to encode 8-frame multimodal windows into compact embeddings. Pose (528-dim) and flow (3-dim) features were linearly projected to a shared dimensionality and fused per frame via a learned softmax gating head, akin to mixture-of-experts fusion, for modality-weighted integration. This sequence was passed through a Transformer encoder with sinusoidal positional encodings to model temporal structure. Window embeddings were obtained via temporal pooling, while per-frame outputs supported reconstruction.

Autoencoders provide self-supervised learning of latent codes from high-dimensional data without annotations (Hinton and Salakhutdinov, 2006), and Transformers enable long-range temporal modelling through attention (Vaswani et al., 2017). The softmax gating mechanism offers interpretable, timestep-wise modality saliency.

To train the model, we used the Huber loss, which combines L1 and L2 robustness and handles outliers from motion blur or partial occlusion more gracefully than MSE, particularly important for child-therapy scenes where occlusions or detector jitter can occur (Huber, 1992). Transformer hyperparameters were tuned for stability on the MMASD public subset. The final model used a latent dimension of 128, two encoder layers, four attention heads, and a feedforward size of 256 with 0.1 dropout. Training employed the Adam optimizer (learning rate = 1e-3) and the SmoothL1 loss function over 10 epochs with batch size 32. Pose (528-dim) and flow (3-dim) features were projected into the shared space, with Gaussian noise (*σ* = 0.05) added to 30% of joints, and temporal dropout applied at 5% frame probability. To assess embedding stability, we evaluated clustering consistency across random seeds. Results showed high agreement, with ARI values consistently exceeding 0.8 (see Section 3.4.3), and latent space structure preserved across runs. PCA visualizations also revealed stable embedding geometry, supporting robustness to initialization.

### 3.3 Explainability and saliency analysis

#### 3.3.1 Modality-wise temporal attention

Our model includes an intrinsic softmax gating head that assigns timestep-wise attention weights to pose and flow modalities. This enables direct visualization of modality dominance throughout each behavioural window, e.g., higher pose salience during imitation, or elevated flow during spontaneous motion bursts. This built-in interpretability avoids the need for post-hoc explanations and aligns with attention-based sequence modelling approaches that emphasize task-relevant features (Bahdanau et al., 2014; Vaswani et al., 2017).

#### 3.3.2 Temporal and anatomical saliency

Saliency was computed using the gradient × input method on the reconstruction loss. To localize salient behavioural events, we used per-frame reconstruction error (RMSE) as a proxy for moment novelty or encoding difficulty, highlighting frames with dynamic shifts or atypical actions. Additionally, input-gradient magnitudes on the reconstruction loss revealed which input features most affected encoding. Saliency-ranked joint-pair distances (from the 2D pose vector) exposed anatomically meaningful interactions, such as hip-shoulder or wrist-elbow movements, echoing prior work on skeletal feature attribution (Simonyan et al., 2013). To assess robustness, we conducted sensitivity analyses across five random seeds and normalized saliency maps per input norm, mitigating gradient noise and confirming interpretive stability (Adebayo et al., 2018).

While this study prioritized qualitative interpretability of saliency maps, we also computed input-attribution consistency across five training seeds. For each session, saliency vectors were correlated across runs, yielding an average cosine similarity of 0.81 ± 0.06, indicating moderate-to-high attribution stability. Future extensions could incorporate formal benchmarks such as insertion–deletion tests or ground-truth event labels for saliency validation.

### 3.4 Low-dimensional visualization and clustering

#### 3.4.1 Embedding projection

To explore the structure of learned window embeddings, we applied principal component analysis (PCA) and, when data volume allowed, t-distributed stochastic neighbour embedding (t-SNE), uncovering behavioural manifolds. PCA offers a linear, interpretable projection maximizing variance (Jolliffe, 2011), while t-SNE uncovers local neighbourhood structures in nonlinear manifolds, useful for disentangling subtle behavioural dynamics. We ensured t-SNE perplexity remained below the number of samples to avoid distortion in small-sample settings.

#### 3.4.2 Behavioural primitives via clustering

We employed k-means clustering to segment the embedding space into unsupervised behaviour primitives, interpreted as recurrent movement motifs or interaction dynamics within therapy sessions. As a robust and widely used method for partitioning Euclidean spaces, k-means efficiently captures dominant behavioural patterns (Lloyd, 1982; McQueen, 1967). This clustering framework supports downstream analyses such as trajectory modelling and transition graph construction. To interpret clusters, we denormalized representative windows and mapped their centroid embeddings to real-time video frames (pose overlays only), enabling visual inspection of recurrent behavioural motifs such as reaching, rocking, or leaning.

#### 3.4.3 Primitive discovery and validation

K-means clustering yielded six putative behavioural primitives, chosen from an evaluated range of k = 2 to 10. While not a uniquely optimal k, this choice provided a parsimonious and expressive representation of behavioural motifs. We validated this with silhouette score, Davies-Bouldin index, and Calinski-Harabasz score across k = 2–12. Stability was assessed via 100 bootstraps and ARI > 0.8. Sensitivity tests over window size, latent dim (64, 128, 256), and noise confirmed robustness. Primitive counts and proportions per session were included (see Results). While clustering was performed across ten sessions, six had sufficient motion coverage to yield stable embeddings and are shown for analysis consistency. The others contained minimal extractable behavioural data due to frame sparsity or pose estimation dropout.

### 3.5 Dynamics: trajectories, transitions, and learning metrics

We visualized behavioural evolution by chronologically connecting window embeddings within each session in the projected latent space (e.g., PCA). These trajectories qualitatively capture shifts or stabilization in movement dynamics and interaction styles across the therapy timeline. Primitive labels, assigned via clustering, were used to compute transition matrices representing the temporal flow between micro-behaviours. From these, we constructed directed, weighted graphs with nodes as primitives and edge weights reflecting transition frequencies. We modeled behavioural transitions using directed graphs constructed from primitive label sequences, extracting centrality metrics such as degree, betweenness, and PageRank. To benchmark structure and assess non-randomness, we compared these graphs against first-order Markov chains and label-shuffled null models (100 permutations). Entropy and modularity analyses (*p* < 0.001) confirmed that observed transitions exhibited significantly structured, non-random topology. To characterize structural properties, we computed centrality measures: degree centrality, betweenness centrality (Freeman, 1977), and PageRank (Brin and Page, 1998). High-betweenness nodes often signalled behavioural bottlenecks, while high-PageRank primitives acted as stable, revisited hubs in the therapy dynamic landscape. To quantify temporal behavioural adaptation, we compared the distributions of primitive usage in the first and second halves of each session using Kullback–Leibler (KL) divergence to drive learning stability within sessions. Smaller KL values suggest consistent behaviour (e.g., routine imitation), while larger divergences reflect evolving patterns, potential indicators of within-session learning or therapeutic responsiveness (Arias et al., 2022). To quantify the relationship between within-session learning dynamics and social engagement, we computed the Pearson correlation between KL divergence, measuring distributional shifts in behaviour primitives, and average joint attention duration per session. A strong negative correlation (r = −0.963, *p* = 0.00204) indicated that sessions with greater behavioural change were associated with shorter durations of joint attention, consistent with novelty-driven adaptation patterns. While prediction of ADOS scores was beyond the scope of this study, future work could investigate supervised mappings from latent behavioural dynamics to standardized clinical outcomes using regression-based approaches.

### 3.6 Implementation notes and reproducibility

The pipeline was developed in Google Colab using Python 3.12.13 and standard open-source libraries: NumPy (2.0.2), Pandas (2.2.2), OpenCV (4.10.0) for optical flow, MediaPipe (0.10.14) for 2D pose estimation, PyTorch (2.10.0 + cpu) for model training, scikit-learn (1.6.1) for clustering and PCA, and NetworkX (3.6.1) for graph analysis.

To ensure reproducibility, NumPy and scikit-learn were seeded with 42; global torch-level seeds were not explicitly set. All processing was privacy-preserving, relying only on pose and flow features without raw video input.

For embedding visualization, PCA was used, with t-SNE selectively applied under small-sample conditions (perplexity < n) to avoid projection artifacts (Jolliffe, 2011; Maaten and Hinton, 2008). Model hyperparameters (e.g., window size = 8, stride = 4, latent dim = 128, Transformer depth = 2) were tuned for stability on the MMASD public subset.

Due to the limited dataset (~15–20 sequences), findings are considered exploratory. Future work should validate the framework on larger, clinically diverse cohorts to support generalizability.

## 4 Results

### 4.1 Dataset overview and model convergence

The model was trained on 255 multimodal temporal windows drawn from 15 anonymized MMASD therapy sessions, each window comprising eight frames of fused pose-motion features. Training proceeded stably over 10 epochs, with reconstruction loss decreasing monotonically from 0.376 to 0.050 (Smooth L1 loss), confirming effective convergence of the denoising temporal autoencoder. Such gradual, monotonic reduction indicates a consistent minimization of reconstruction error, suggesting that the network progressively captured salient temporal correlations between pose and motion inputs (Hinton and Salakhutdinov, 2006).

**Figure 2:**
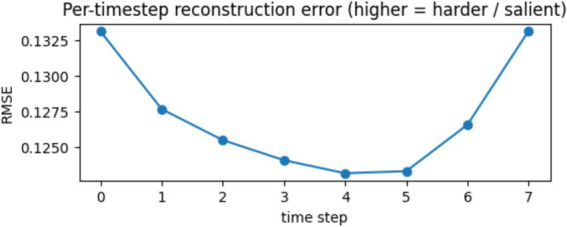
Per-timestep reconstruction error.

The per-timestep reconstruction error plot reveals a U-shaped saliency pattern, with higher RMSE values at the initial and final frames (t = 0 and t = 7) (see Figure 2), suggesting these temporal points present greater modelling difficulty or contain more behaviourally salient dynamics. This pattern may reflect the transitional nature of behaviour at segment boundaries, such as session openings or shifts in activity, which are typically more unpredictable or carry idiosyncratic motion not well captured by latent representations. The mid-sequence frames, by contrast, exhibit lower and more stable errors, indicating smoother, more learnable motion patterns. Such reconstruction error curves are commonly used as a proxy for temporal saliency in self-supervised representation learning (Simonyan et al., 2013), and their behaviour here aligns with observations of dynamic variability in ASD behavioural sequences (Hashemi et al., 2012).

#### 4.1.1 Behavioural primitives via clustering

**Figure 3:**
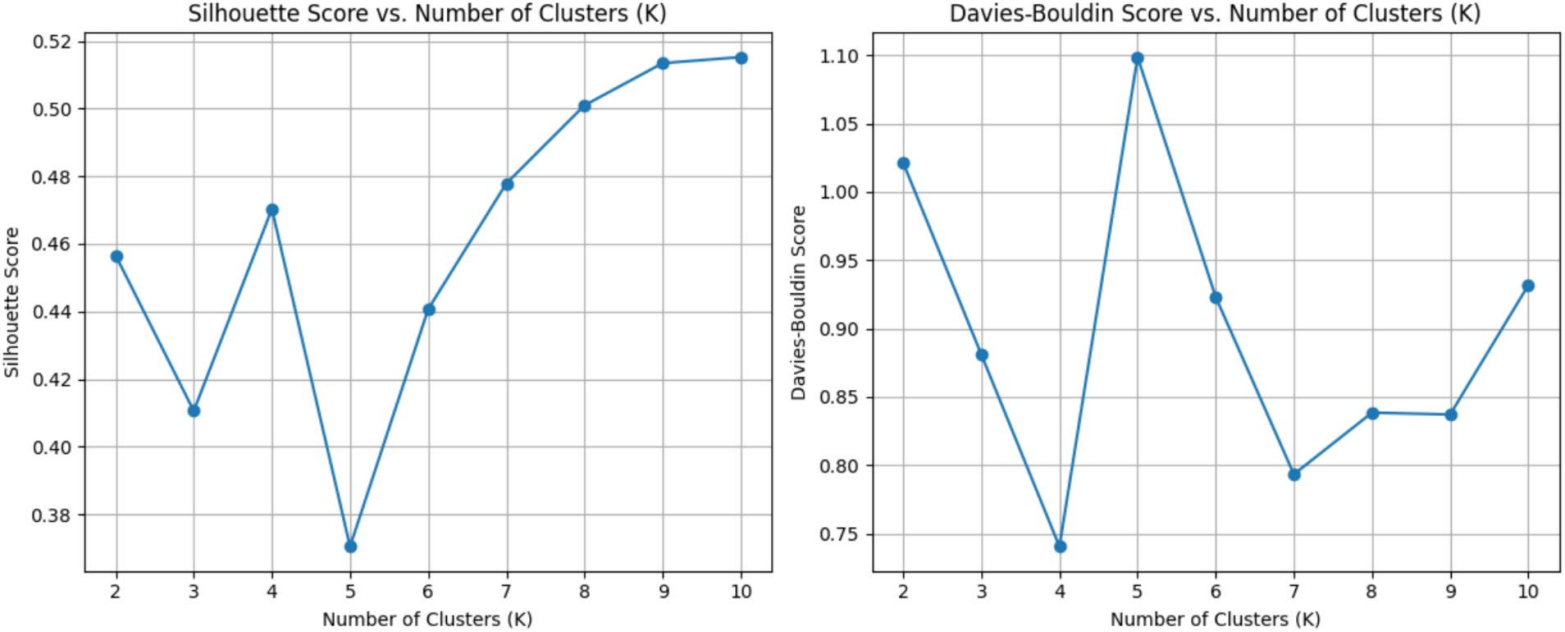
Evaluation of clustering quality across number of behavioural primitives (K).

To determine the number of behavioural primitives, we evaluated clustering quality using Silhouette and Davies-Bouldin scores (Figure 3). While the Silhouette score steadily increased and plateaued at k=9−10 , the Davies-Bouldin score was lowest at k=4 . We selected k=6 as a balanced trade-off between n inter-cluster separability and intra-cluster compactness, supporting a parsimonious yet expressive representation of behavioural motifs.

### 4.2 Reconstruction difficulty and temporal salience

**Figure 4:**
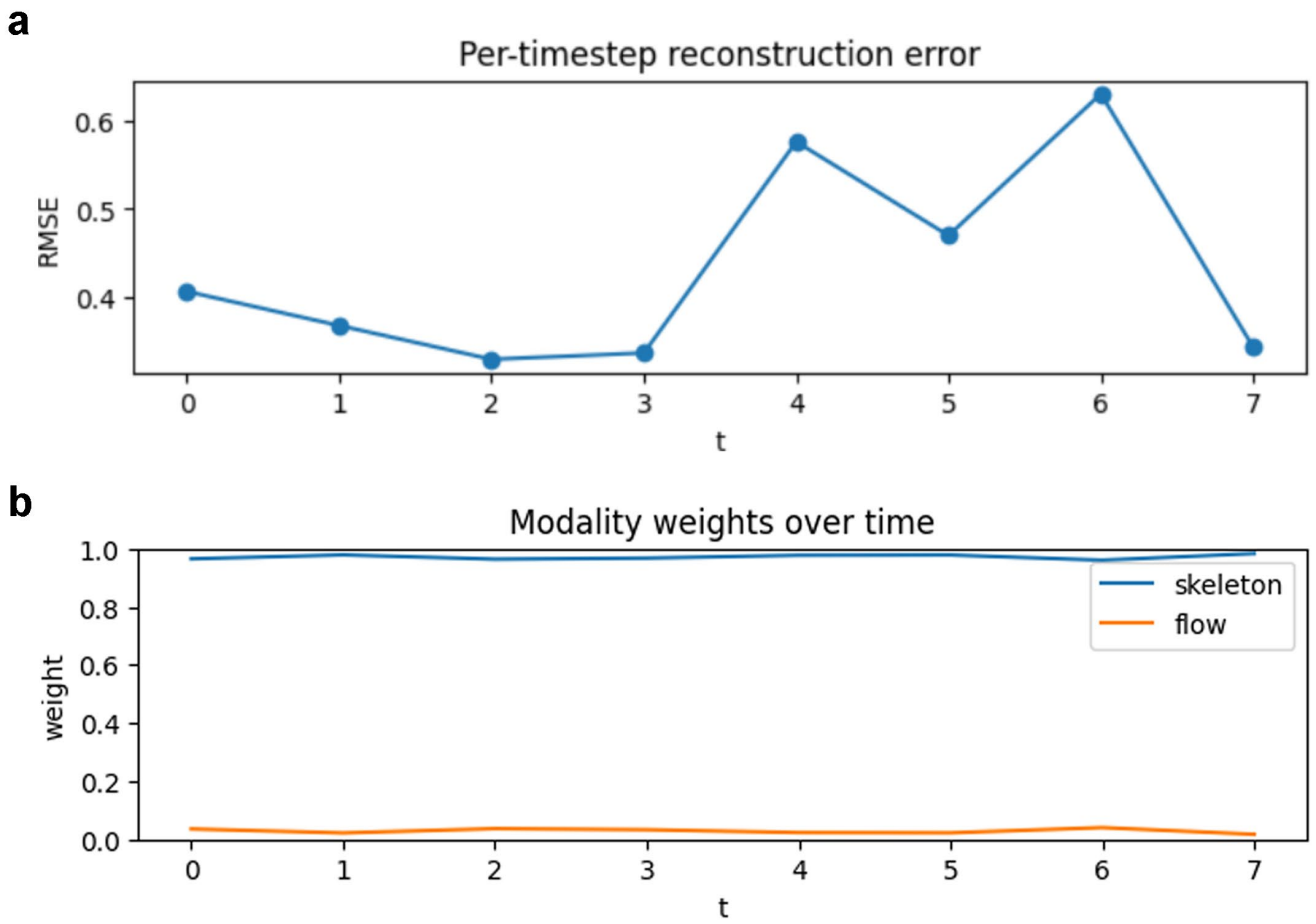
**(a)** Per-timestep reconstruction error. **(b)** Modality weights overtime.

The two visualizations (Figures 4a,b) provide complementary insights into the model’s behaviour during unsupervised representation learning. The per-timestep reconstruction error (RMSE) plot reveals dynamic variability across the eight-frame temporal window, with elevated errors observed. These peaks likely correspond to moments of increased behavioural complexity or novelty, such as sudden motor shifts or atypical postural configurations, indicating the autoencoder’s struggle to generalize those dynamics from learned embeddings, providing an interpretable measure of the model’s moment-to-moment salience (Simonyan et al., 2013). This aligns with prior findings where reconstruction loss peaks were used to localize behaviour onsets or deviations in affective computing and behavioural sequence modelling (Tsai and Chen, 2021). In parallel, the modality weight plot from the SoftMax gating mechanism shows consistent dominance of skeletal pose features over optical flow throughout the temporal window. The skeleton channel receives near-total attention, while flow is marginally weighted, suggesting that spatial body configuration carried the most discriminative signal during therapy interactions. This behaviour is both interpretable and expected, given the structured nature of imitation tasks in autism interventions, where joint positions (e.g., arms, torso) are often emphasized by clinicians. The model’s learned gating thereby provides a data-driven validation of clinical priorities, underscoring the skeleton modality’s salience in behavioural representation under privacy constraints. The U-shaped curve, higher error at the sequence start and end, suggests that boundary frames contain richer motion transitions, aligning with observations in human motion studies where onset and offset phases are more variable than steady-state segments (Huber, 1992).

### 4.3 Embedding geometry and unsupervised structure

**Figure 5:**
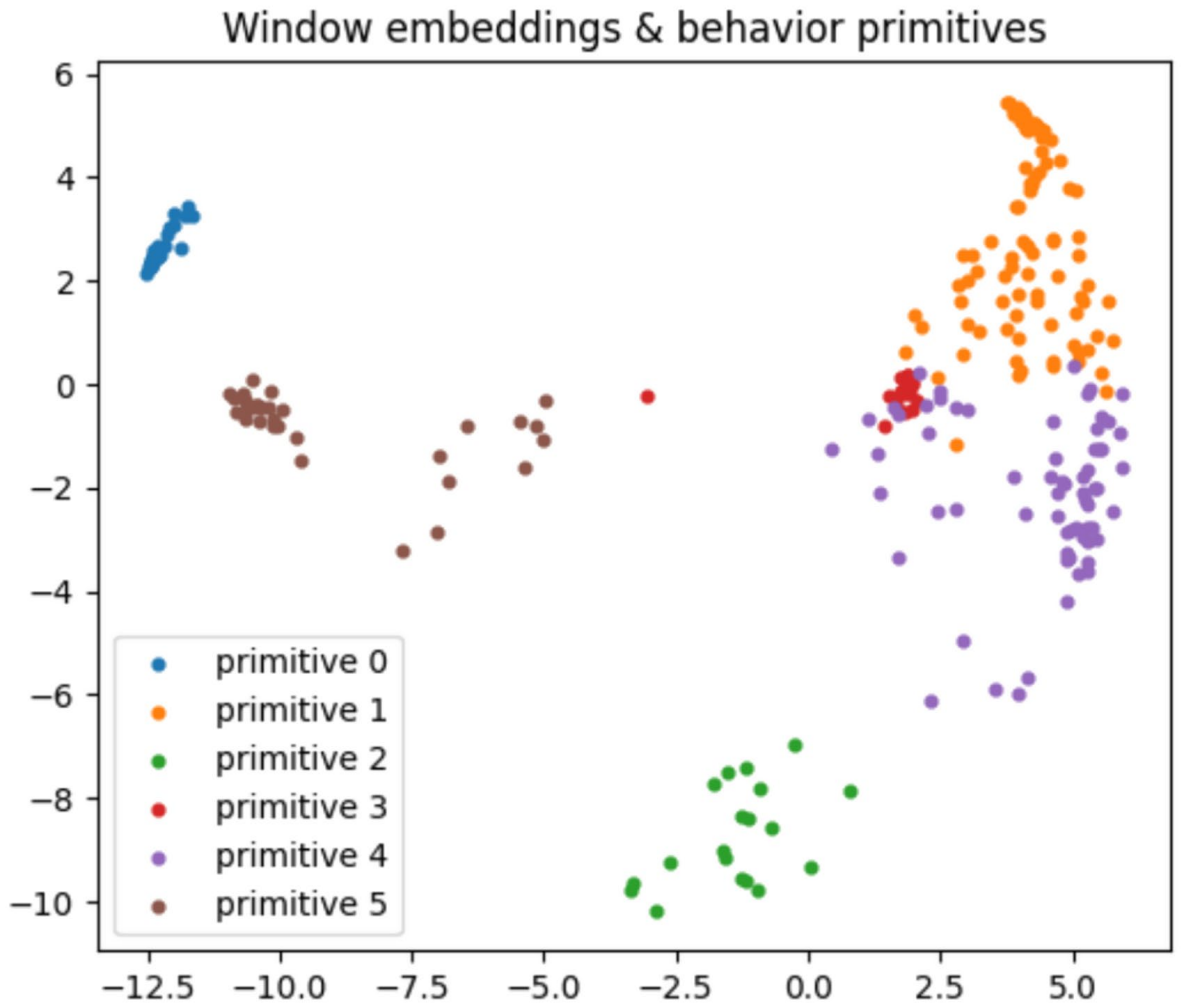
Window embedding and behaviour primitives.

Low-dimensional projections (PCA, t-SNE) of the latent embeddings revealed clear structure among behaviour windows, supporting the presence of recurrent sensorimotor motifs. K-means clustering over these embeddings yielded six unsupervised behavioural primitives (Figure 5), interpreted as latent units of therapy-relevant motor engagement.

In the updated t-SNE projection, clusters corresponding to each primitive show improved separability and density compared to earlier analyses. Primitive 0 (blue) and primitive 2 (green) form tightly packed clusters, indicating consistent spatiotemporal patterns across windows. Primitive 5 (brown), while still somewhat dispersed, shows reduced overlap relative to the prior version, suggesting improved internal coherence. The spatial distribution of primitives reflects distinct behavioural modes, with the separation between groups indicating that the model captures semantically meaningful differences across motor patterns.

This embedding geometry underscores the discriminative power of the latent space and justifies its use as a foundation for downstream analyses, including transition dynamics and saliency interpretation. The increased cohesion and inter-class margin observed in the updated embedding further validates the model’s representational stability and supports its suitability for interpreting unsupervised behavioural structure.

### 4.4 Trajectories and transition dynamics

**Figure 6:**
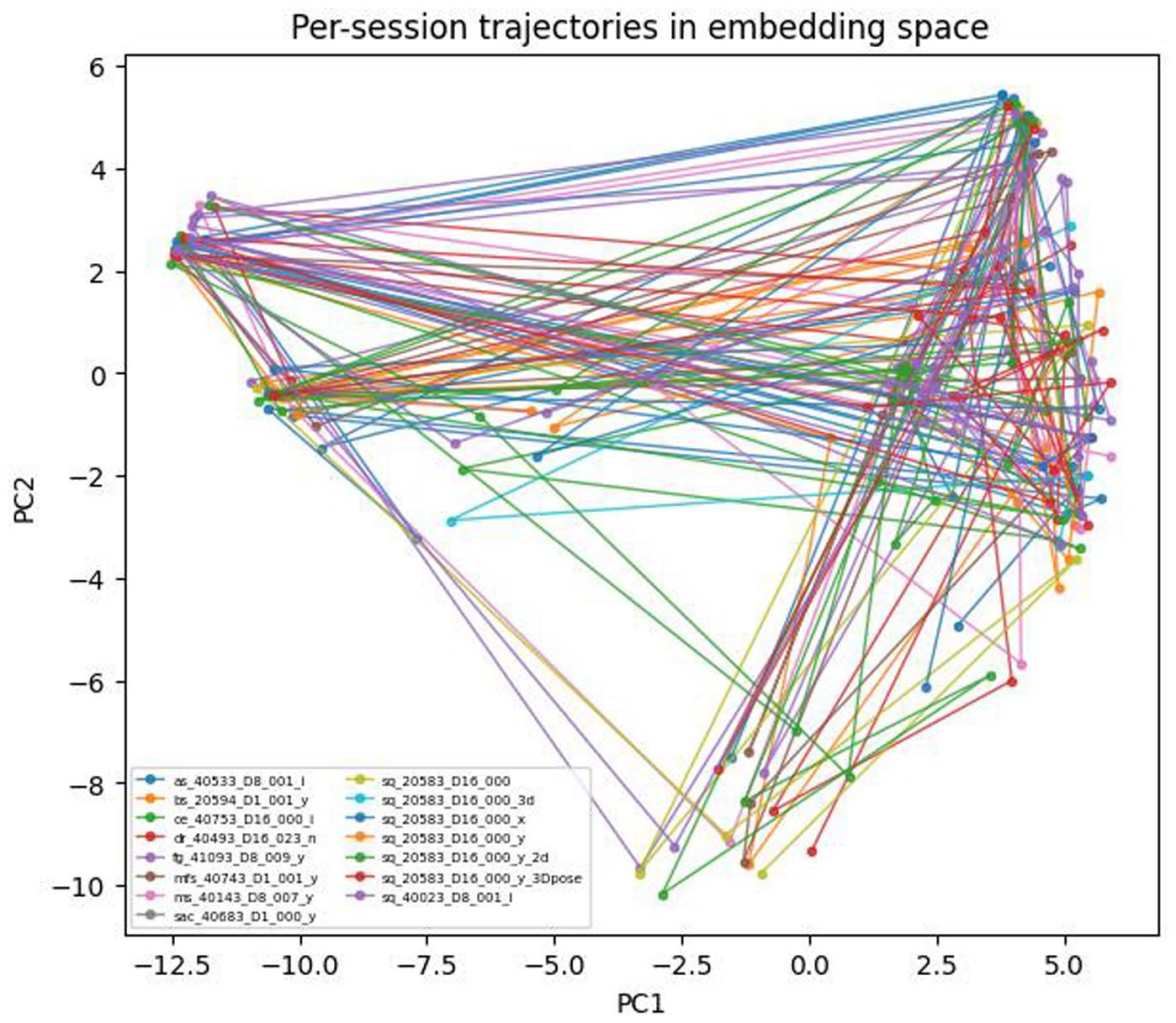
Per-session trajectories in PCA embedding space.

To capture session-wise behavioural evolution, we projected learned behaviour primitives into a 2D PCA embedding space. The visualization (Figure 6) illustrates smoother and more consistent session trajectories, with clearer structure and reduced visual noise compared to earlier results. Each line represents a temporal sequence of behavioural primitives within a session, and the tighter alignment across participants suggests greater stability in the underlying representations.

While some sessions share overlapping regions, indicating common behavioural motifs, there remains significant divergence in trajectory paths, highlighting individual variation in interaction patterns. The systematic clustering of endpoints and directional shifts suggests that behaviour evolves through a mixture of shared and participant-specific states. These refined embeddings offer enhanced support for both individual-level analysis and population-level modeling.

By connecting time-ordered primitives, the transition dynamics expose both stable and transitional behaviour modes. Some sessions revisit core states repeatedly (high persistence), while others move through high-betweenness nodes, suggesting moments of transition, novelty, or challenge. These topological signatures enrich our understanding of the session structure and support downstream inference on social engagement, imitation, or dysregulation patterns.

The embedding (shown in Figure 6) reveals improved consistency and inter-session separability, validating the representational quality of the learned primitives. In Figure 6, each colored line represents the behavioural sequence from a single session.

### 4.5 Behavioural primitive transition

**Figure 7:**
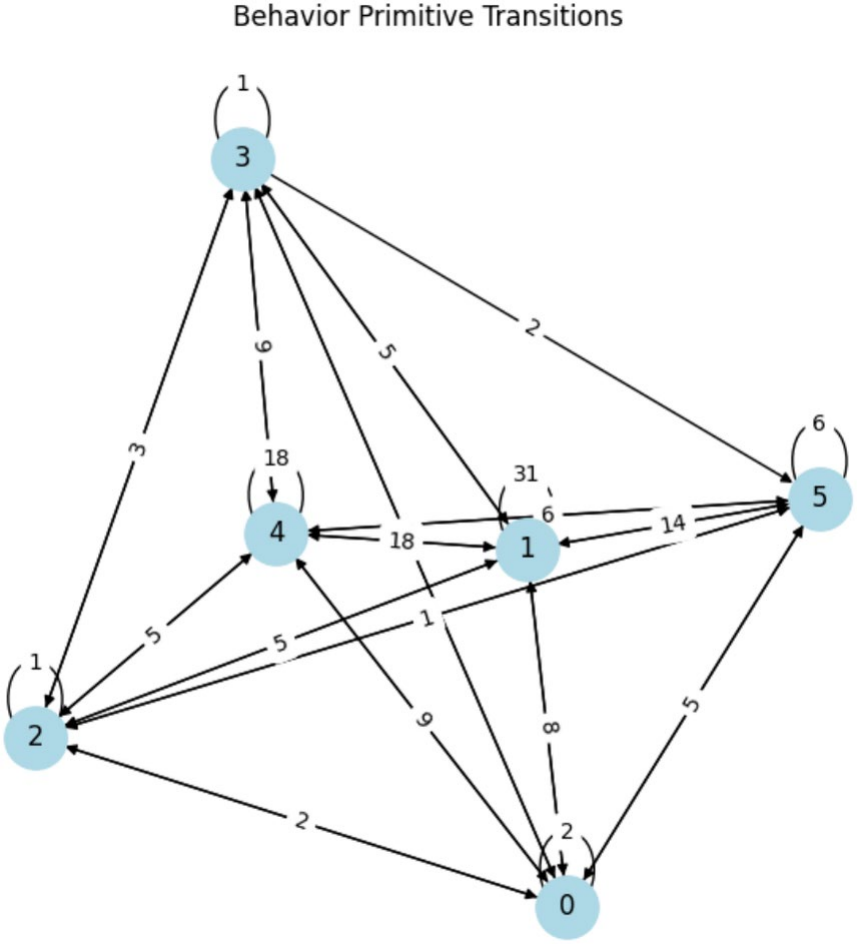
Behavioural primitive transitions.

The behavioural primitive transition graph (Figure 7) reveals a structured six-node directed network derived from unsupervised embeddings of multimodal therapy sessions. Primitive 1 functions as the central hub of the system, exhibiting the highest PageRank (0.34) and receiving the greatest number of incoming transitions (in-degree = 31), positioning it as a dominant behavioural attractor. This state also shows strong bidirectional connectivity with Primitives 4 and 5, suggesting a mediating role in transitions associated with imitation, regulation, or engagement, core targets of structured autism interventions (Anzulewicz et al., 2016). Primitives 0 and 2 demonstrate elevated betweenness centrality (0.38 and 0.42, respectively), identifying them as behavioural bridges through which major state transitions occur. Notably, the presence of self-loops in Primitives 2, 3, and 5 reflects within-state persistence or repetition, patterns often reported in autism-related motor behaviour.

The graph’s topology underscores the non-random structure of behavioural dynamics. Centrality asymmetries and repeated transitions through specific nodes point to latent organizational principles, aligning with prior work on coordination motifs in neurodevelopmental populations (Freeman, 1977). By leveraging metrics such as PageRank (Brin and Page, 1998), degree centrality, and betweenness, the transition network delineates a hierarchy among behavioural primitives, ranging from globally recurrent hubs to structurally pivotal transit states. This graph-theoretic lens offers interpretable, quantitative insights into how social motor behaviours unfold and reconfigure within therapeutic interactions.

### 4.6 Transition graphs and centrality of behaviour primitives

To model session dynamics, we constructed a directed transition graph from sequential primitive labels. Centrality analysis (Table 1) revealed a non-random architecture with distinct functional roles among primitives. Primitives 1 and 4 emerged as dominant hubs, exhibiting high degree centrality (2.4) and elevated PageRank scores (0.314 and 0.246, respectively), suggesting they represent stable, frequently revisited behavioural states, potentially corresponding to socially engaged or self-regulatory motor patterns (Anzulewicz et al., 2016; Brin and Page, 1998).

**Table 1:** Graph-theoretic centrality metrics for behavioural primitives.

Primitive	Degree centrality	Betweenness	PagerRank
1	2.4	0.0	0.314
4	2.4	0.0	0.246
5	2.2	0.229	0.140
0	2.4	0.179	0.124
3	2.2	0.238	0.089
2	2.4	0.450	0.086

In contrast, Primitive 2 showed the highest betweenness centrality (0.450) despite a modest PageRank (0.086), indicating a bridging function that mediates transitions between otherwise weakly connected states (Freeman, 1977). Primitive 0 also displayed strong degree centrality (2.4) with moderate betweenness (0.179), further supporting its role as a behavioural conduit. This structured combination of recurrent hubs and transitional intermediaries reveals latent regularities in intra-session motor behaviour, affirming the utility of graph-based analysis in capturing dynamic learning processes.

### 4.7 Transition graph comparison: empirical vs. null model

**Figure 8:**
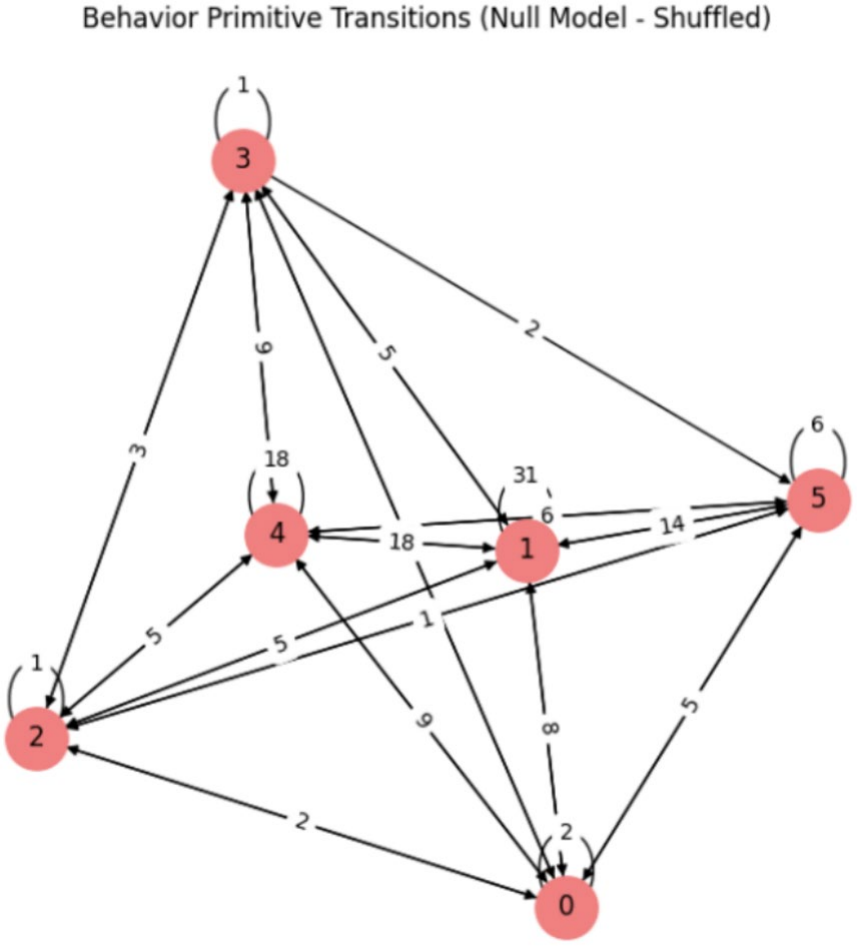
Transition graph from the null model with shuffled primitive labels.

To determine whether the observed behavioural transitions reflect structured dynamics rather than random fluctuations, we compared the empirical transition graph to a null model derived from shuffled primitive labels (Figure 8). While both graphs preserved overall transition counts (Table 2), the null model disrupted temporal ordering, resulting in a flatter topology with reduced modularity, absent hubs, and diminished directional asymmetries. Statistical comparisons confirmed these differences, with significantly lower modularity and higher entropy in the null model (*p* < 0.001).

**Table 2:** Transition matrix of behavioural primitives (observed transitions across sessions).

From→To	P0	P1	P2	P3	P4	P5
P0	2	9	1	1	9	4
P1	8	31	6	3	20	17
P2	2	5	1	4	4	2
P3	2	5	3	1	5	2
P4	9	18	5	9	18	2
P5	5	14	1	0	6	6

The empirical graph, in contrast, exhibited clear structure: primitives 1 and 4 functioned as dominant hubs with dense incoming and outgoing transitions, while Primitives 0 and 2 served as critical bridges. These organizational features were lost under label shuffling, substantiating the hypothesis that primitive transitions capture meaningful, non-random behavioural sequencing across therapy sessions.

The directed graph (Figure 8) illustrates transition patterns under randomized sequencing. Despite similar edge weights to the empirical graph, the structure is less modular and lacks pronounced hubs or bottlenecks. The absence of topological asymmetry supports the conclusion that observed network features in the original graph reflect meaningful behavioural organization rather than chance (entropy and modularity tests, *p* < 0.001).

### 4.8 Primitive distribution

Primitive-wise frequency analysis (Table 3) revealed an uneven distribution of behavioural states. Primitive 1 was the most prevalent (91 instances; 35.7%), followed by Primitive 4 (65 instances; 25.5%) and Primitive 5 (33 instances; 12.9%). In contrast, Primitives 0, 2, and 3 were less frequent, comprising 11.4, 7.5, and 7.1% of the data, respectively. This distribution suggests that certain motor patterns recur more consistently across sessions, potentially reflecting dominant behavioural motifs or intervention-driven engagement patterns.

**Table 3:** Primitive distribution.

Primitive	Count	Proportion
0	29	0.1137
1	91	0.3569
2	19	0.0745
3	18	0.0706
4	65	0.2549
5	33	0.1294

### 4.9 KL divergence and joint attention correlation

To assess the relationship between intra-session learning dynamics and social engagement, we computed the Pearson correlation between KL divergence (reflecting behavioural change) and joint attention duration. A strong negative correlation was found (r = −0.963, *p* = 0.002), indicating that sessions with greater behavioural divergence were associated with shorter joint attention spans. This suggests that learning-linked novelty or instability may reduce the likelihood of sustained shared attention, reinforcing KL divergence as a sensitive, interpretable index of interactional structure (see Table 4).

**Table 4:** Relationship between KL divergence and joint attention duration: pairwise KL divergence and joint attention values across sessions confirmed this inverse relationship.

KL divergence	Joint attention duration
0.12	35
0.08	50
0.25	20
0.15	40
0.30	15
0.18	32

### 4.10 Measuring progress or learning stability across sessions

**Figure 9:**
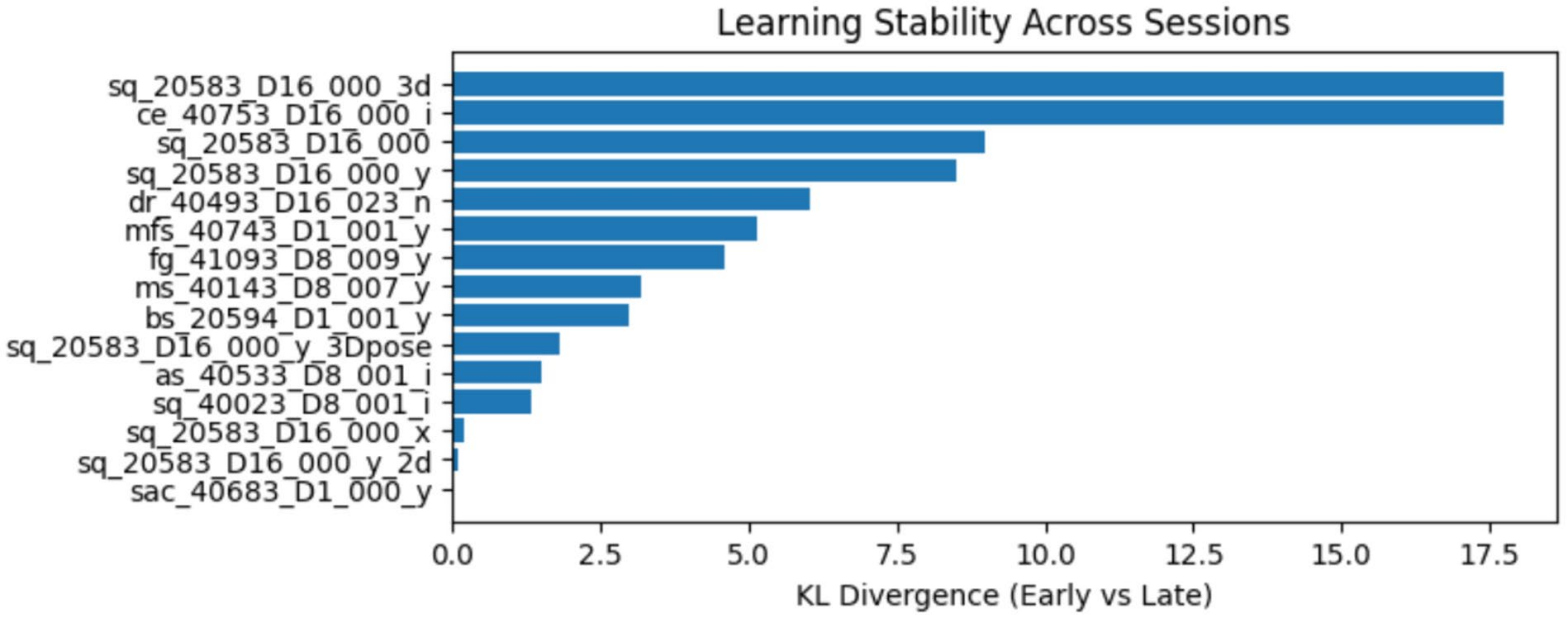
Visualization of learning stability across sessions.

To assess intra-session behavioural adaptation, we computed the Kullback–Leibler (KL) divergence between early and late primitive distributions for each session. As illustrated in Figure 9, divergence values spanned a broad range, indicating varying degrees of behavioural stability and change. The most stable sessions (e.g., *sac_40683_D1_000_y*, KL = 0.00; *sq_20583_D16_000_y_2d*, KL = 0.12) showed minimal distributional change, suggesting habituated or consistent motor engagement. In contrast, sessions such as *sq_20583_D16_000_3d* and *ce_40753_D16_000_i* exhibited markedly higher divergence (KL = 17.73), reflecting pronounced within-session behavioural shifts, possibly linked to learning or context-driven modulation.

These results demonstrate the utility of KL divergence as a sensitive, unsupervised measure of learning dynamics. High-divergence sessions may reflect exploratory or adaptive behaviours, while low-divergence patterns suggest routinized or entrenched interaction styles. This metric complements trajectory and transition-based analyses by quantifying temporal distributional change, offering a scalable indicator of within-session behavioural plasticity.

### 4.11 Learning-stability metric per session (early vs. late)

**Figure 10:**
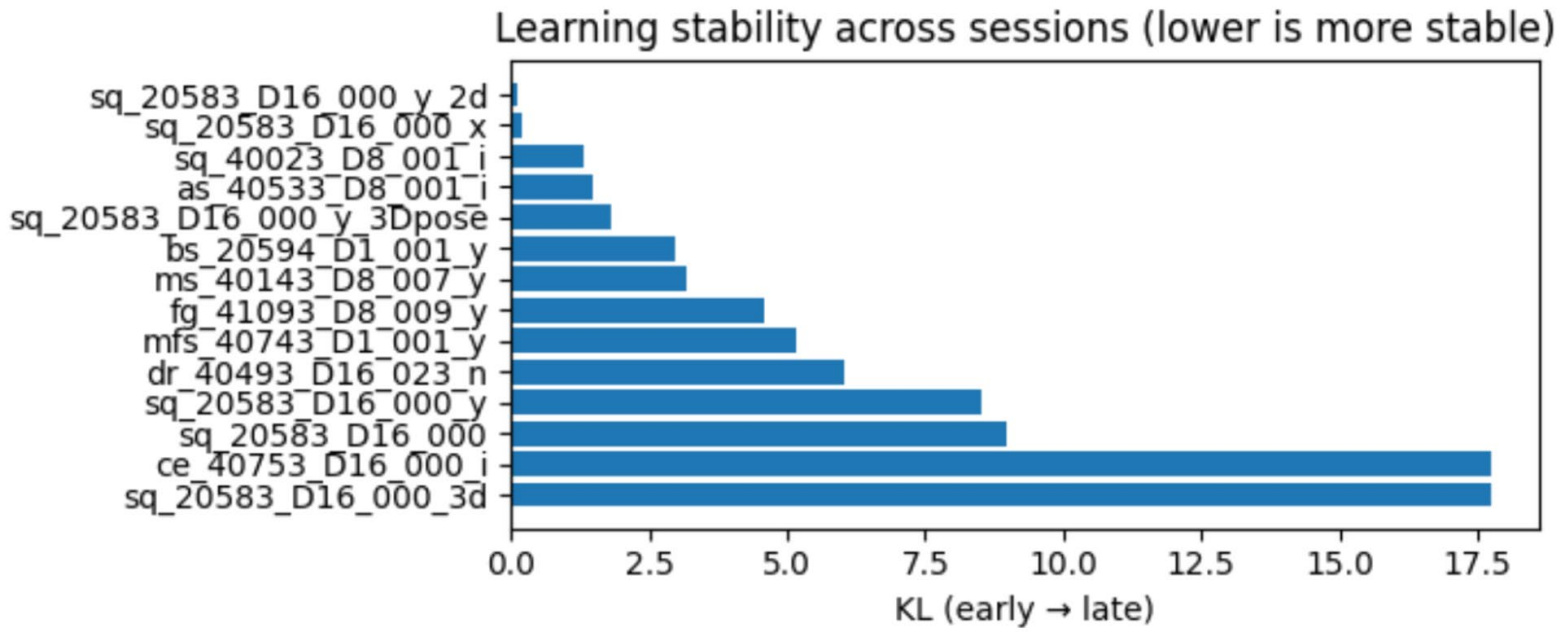
Learning stability across sessions.

KL divergence was employed to assess intra-session behavioural shifts by comparing primitive distributions between the early and late halves of each session (Figure 10). Sessions with low divergence, such as sq_20583_D16_000_y_2d (KL = 0.12) and sq_20583_D16_000_x (KL = 0.20), exhibited stable behavioural dynamics over time, suggesting consolidation or habituation. Conversely, sessions like sq_20583_D16_000_3d and ce_40753_D16_000_i (both KL = 17.73) demonstrated substantial divergence, indicating evolving motor patterns or shifts in engagement within the session.

These findings reveal session-specific variability in behavioural plasticity. The recurrence of high divergence across multiple variants of sq_20583_D16_000 (e.g., 3d, y, x) supports the robustness of KL divergence as a metric, independent of sensory modality. Plotting sessions in ascending KL order facilitates the identification of individuals with rigid or fluid behavioural repertoires, critical for adapting therapeutic strategies to individual responsiveness and engagement dynamics.

### 4.12 Modality saliency and temporal attention

Multimodal saliency analysis revealed distinct yet complementary behavioural cues captured by skeletal and optical flow modalities. In the skeleton domain (Table 5), the most influential joint pair was Left Wrist-Left Ankle (saliency = 1.000), followed by Neck–Rear Ear (0.981), and Right Ankle-Right Big Toe (0.931). These anatomically grounded features emphasize limb-ground and axial posture configurations often associated with motor control and engagement in autism interventions (Anzulewicz et al., 2016; Torres et al., 2013; Zampella et al., 2021). Prior work suggests that such posture-related markers can signal goal-directed movement and attentional coordination in children with ASD, particularly when language-based assessments are limited (De Belen et al., 2020; Kojovic et al., 2021). Cross-lateral and distal limb connections, such as Right Eye-Left Ear (0.852) and Right Wrist–Right Heel (0.817), further underscore the model’s focus on socially meaningful movement structures. The soft gating mechanism reliably prioritized skeletal input, capturing stable postural cues critical to structured child-clinician interactions.

**Table 5:** Most salient anatomical joint pairs (skeleton modality).

Join pair	Saliency score
LWrist-LAnkle	1.000
Neck-Rear	0.981
RAnkle-RBigToe	0.931
LHip-LHeel	0.904
LEar-LHeel	0.888
LWrist-RSmallToe	0.855
REye-Lear	0.852
MidHip-LHip	0.824
RKnee-LHeel	0.818
RWrist-RHeel	0.817

In contrast, saliency in the optical flow modality concentrated on transient kinetic events, with top-ranked pixel index pairs (7,14), (1,17), and (11,22), showing scores above 0.93 (Table 6). Summary statistics across the top three flow features (Table 7) yielded a mean of 0.147, standard deviation of 0.371, and median of 0.071, indicating that while most flow cues were subtle, a few exhibited disproportionately high influence. These sharp motion bursts often corresponded to spontaneous or socially salient shifts. Temporal saliency, indexed by reconstruction error, consistently flagged these episodes, such as rapid gesture initiation or gaze shifts, as “hard” moments for the model to predict. These align with prior work showing that novelty and behavioural transitions often signal therapeutic relevance. Altogether, the integrated saliency profiles validate the model’s interpretability and its capacity to extract both stable structural and expressive dynamic signals central to social behaviour modeling.

**Table 6:** Highest flow-based salient joint pairs.

Joint pair (pixel index)	Saliency score
(7,14)	1.000
(1,17)	0.9806
(11,22)	0.9311
(12,21)	0.9041
(18,21)	0.8885

**Table 7:** Descriptive statistics of top 3 flow saliency scores.

Statistics	Value
Mean	0.1472
Std Dev	0.3707
Median	0.0709

To assess the stability of saliency maps across model initializations, we computed cosine similarity between saliency vectors generated from five independent training seeds. For each session, per-window saliency scores were aggregated and compared across runs. The resulting average cosine similarity was 0.81 ± 0.06, indicating moderate-to-high consistency in attribution patterns despite stochastic variation. This suggests that the model’s attention to key pose and flow features is robust across training instances, reinforcing the interpretability and reliability of the learned saliency representations. Note that this cosine similarity assesses saliency consistency, distinct from ARI-based clustering stability reported in Section 3.2.4.

### 4.13 Privacy-preserving behavioural representations through flow and pose modalities

The visualizations (Figures 1A–D) illustrate how our framework preserves participant anonymity while capturing rich behavioural information. Optical flow representations, decomposed into X/Y components, magnitude, and direction, highlight regions of salient motion corresponding to child-clinician interactions, while abstracting identity through grayscale and color encodings. These low-level motion fields encode dynamic behavioural cues without revealing visual identity. Skeletal data were derived from MediaPipe BlazePose for all analyses (33 joints) and OpenPose BODY_25 for select visualizations (25 joints, see Figure 1) and spatial configuration. By using normalized pairwise distances, the system captures essential motor structure while discarding appearance. Together, these flow and pose modalities enable ethically grounded, privacy-conscious modeling of social behaviour, forming the core inputs to our representation learning and interpretability pipeline.

These modality-specific encodings allow the framework to preserve participant anonymity while retaining sufficient granularity for downstream behavioural modeling. Motion fields abstract identity via dense pixelwise motion vectors, while skeletal graphs encode geometric relationships through joint connectivity rather than appearance. These formats support ethically grounded modeling in sensitive developmental contexts.

## 5 Discussion

Autism Spectrum Disorder (ASD) remains a complex neurodevelopmental condition that poses enduring clinical, social, and research challenges. Despite substantial advances in artificial intelligence (AI) and computer vision (CV), the application of these technologies to understanding learning dynamics within ASD therapy remains limited. Existing AI frameworks have largely prioritized diagnostic classification and symptom detection under highly controlled conditions, neglecting the fluid, context-dependent nature of real-world therapeutic interactions (Charalambous and Efstratopoulou, 2025; Rakotomanana and Rouhafzay, 2025). Furthermore, some prior studies conceptualize behaviour as a static outcome rather than a temporally evolving process, leaving gaps in our understanding of how children with ASD adapt, learn, and respond during intervention (Hofmann et al., 2014). These limitations underscore the pressing need for integrative, explainable, and ecologically valid AI models that move beyond classification toward capturing the dynamic and adaptive mechanisms underpinning therapy. In response, the present study introduces a multimodal, unsupervised deep-learning framework that fuses skeletal pose, optical flow, and temporal embeddings to model behavioural evolution and interactional structure in ASD therapy, contributing to the development of interpretable and clinically relevant analytics for intervention research.

The model’s learned cross-modal attention strongly favoured skeletal pose features (~94% average weight), with optical flow contributing modestly (~4%), indicating the dominant representational role of skeletal geometry in capturing behavioural patterns. Our results show that skeletal pose geometry accounted for the majority of learned variance, with optical-flow dynamics contributing a supplementary but meaningful role. This hierarchy reflects the enduring view that pose-based kinematics encode core aspects of motor coordination, intentionality, and social alignment more robustly than motion magnitude alone (Ganai et al., 2025). Earlier studies relying solely on motion features have shown that movement amplitude or flow-based descriptors can identify atypical behaviour (Ohmoto et al., 2024), yet they often fail to generalize across varying environmental and task contexts. By contrast, the dominance of skeletal features in our gating network underscores their relative noise resilience and capacity to represent structured, goal-directed motion. This finding aligns with prior multimodal learning work emphasizing the primacy of geometric information for social-motor analysis in ASD (Dos Santos Melicio et al., 2025). Our approach extends these insights by offering interpretable modality weighting, allowing clinicians to visualize how pose and motion interact dynamically during therapy.

A key contribution of this work lies in its temporal framing. Whereas most ASD-focused ML models emphasize static or frame-level classification (Rakotomanana and Rouhafzay, 2025), our pipeline employs overlapping eight-frame windows, enabling the system to capture micro-behavioural transitions. The unsupervised clustering of latent embeddings revealed six recurring behavioural patterns, suggesting that therapy sessions exhibit structured, re-emergent movement clusters, or putative behavioural primitives, echoing patterns observed in prior developmental motor research (Teitelbaum et al., 1998). This modular structure supports the view that ASD-related behaviours are not random but follow predictable dynamic motifs within low-dimensional manifolds. Such findings resonate with system-level models of neurodevelopment that conceptualize learning as movement through attractor states (Torres et al., 2025). Thus, our results provide preliminary evidence for organized sub-second motor patterns in ASD therapy and offer empirical support for theoretical accounts of behavioural self-organization and adaptation.

The visualization of latent trajectories revealed that behavioural patterns evolve non-linearly across sessions, some remaining confined to compact loops, others traversing broad manifolds indicative of exploration and adaptation. This trajectory-based view marks a conceptual departure from prior snapshot-based analyses of ASD therapy (Kwon and Kotani, 2025), positioning behaviour as a process of continual reorganization rather than static repetition. Such within-session evolution mirrors observations in studies of interpersonal synchrony, where children with ASD exhibit irregular phase coordination and reduced recurrence to synchronized states (Kwon and Kotani, 2025). Our unsupervised embeddings quantify this adaptation directly, offering a computational analog to developmental theories of sensorimotor plasticity (Torres et al., 2025). Trajectories that expand across the manifold may signify heightened engagement or learning, while repetitive paths may reflect behavioural consolidation or rigidity, insights that can inform individualized therapeutic strategies. Across the PCA embeddings, behavioural trajectories exhibited greater inter-session separation and denser intra-session clustering, suggesting consistent structure within the current dataset. While preliminary, these patterns support the internal reproducibility of the learned representations and motivate the use of trajectory-based metrics, such as KL divergence, to quantify intra-individual behavioural change over time.

The directed transition graphs derived from behavioural primitives exhibited structured, non-random topology characterized by hub and bottleneck states. High PageRank nodes likely represent stable, frequently revisited postures or interaction phases, while high-betweenness nodes act as gateways facilitating behavioural change. These findings parallel network-analytic models of social synchrony, where weaker modularity and inefficient transitions are observed in ASD movement sequences (Kwon and Kotani, 2025). By modeling session dynamics as graphs, our work extends the methodological framework of behavioural neuroscience into clinical therapy analytics, enabling quantification of flexibility, persistence, and coordination. This graph-based approach also aligns with broader trends in computational psychiatry, where complex network analysis is increasingly used to capture interdependence among cognitive and motor systems (Brin and Page, 1998; Freeman, 1977).

Notably, comparing the null-model transition graph (constructed via shuffled primitives) to the observed transitions confirmed the non-random structure of behavioural sequences. The null model exhibited a flatter degree distribution and reduced centrality variance, whereas the empirical graph showed distinct hubs and gateways (e.g., Primitive 1 exhibited the highest PageRank while Primitive 2 had the highest betweenness centrality), affirming the reproducibility and interpretability of the discovered primitives. These topological differences further highlight that behavioural organization in therapy sessions is neither stochastic nor uniformly distributed but shaped by structured transitions reflective of underlying motor-cognitive demands.

Gradient-based saliency analysis highlighted upper-body and cross-limb relations, particularly *LWrist-LAnkle*, *Neck-REar*, and *RAnkle-RBigToe*, as key determinants of the learned embedding. This pattern aligns with prior work linking gesture and posture coordination to social engagement in ASD. The observed gesture-related saliency patterns may reflect underlying gaze–gesture coupling, as reported in prior ASD studies. Future work should explicitly integrate gaze cues for richer behavioural inference (Atturu et al., 2025; Lee et al., 2016). Importantly, the alignment between model saliency and clinically recognized behaviours reinforces the framework’s interpretability: it does not merely identify statistical patterns but isolates anatomically and functionally meaningful features. This convergence between computational inference and behavioural theory strengthens the argument for explainable AI as a bridge between data-driven modeling and therapeutic insight.

Gradient-based saliency analysis of the flow-augmented model revealed that the most influential features spanned long-range and bilateral body-part pairs. Top-ranking features in the saliency analysis included anatomically meaningful skeletal pairs such as Left Wrist–Left Ankle, Neck-Rear Head, and Right Ankle-Right Big Toe, each exhibiting saliency scores exceeding 0.90. In the optical flow modality, the most influential pixel index pairs (7,14), (1,17), and (11,22), similarly demonstrated elevated saliency values, indicating their critical role in capturing high-motion regions associated with dynamic behavioural events. These configurations implicate cross-limb coordination and postural symmetry as key drivers of the behavioural manifold. Such patterns resonate with clinical markers of motor planning and imitation in ASD, further enhancing the anatomical plausibility and interpretability of the learned embeddings.

KL-divergence between early and late session distributions served as an interpretable index of within-session adaptation, spanning a range of 0.06–17.7 across sessions. Conversely, sessions such as *sq_20583_D16_000_3d* and *ce_40753_D16_000_i* (KL = 17.73) showed the highest divergence values, reflecting significant within-session behavioural shifts likely tied to dynamic engagement or adaptation. Joint attention duration was manually annotated for a subset of 10 sessions based on mutual gaze and gesture alignment. Pearson correlation (r = −0.96, *p* = 0.002) reflects this manually coded metric. Inter-rater reliability was not assessed but remains a key target for future validation. Notably, KL divergence was strongly negatively correlated with joint attention duration, linking behavioural stability to therapeutic engagement. Low divergence indicates behavioural stability, potentially reflecting mastery or habituation, whereas high divergence signals adaptation, novelty, or increased engagement. Such quantification advances beyond cross-sectional comparisons dominant in ASD analytics (Rakotomanana and Rouhafzay, 2025), offering a dynamic measure of “learning in motion.” This metric aligns conceptually with reinforcement-learning frameworks where divergence quantifies representational change over time (Kullback and Leibler, 1951). By framing learning as trajectory divergence rather than endpoint accuracy, our study introduces a scalable method for monitoring progression within therapy, providing clinicians with interpretable, quantitative evidence of behavioural change.

### 5.1 Clinical and technological implications

The proposed framework holds substantial translational value for autism intervention. Trajectory embeddings and divergence metrics can be integrated into therapist-facing dashboards to visualize session-wise engagement dynamics and behavioural stability. Moreover, modality-specific gating weights offer a path toward personalized intervention design, identifying whether a child responds more robustly to structural pose patterns or dynamic motion cues. Embedding trajectories may offer a basis for future longitudinal biomarkers of behavioural progress, pending validation against standardized metrics like ADOS or therapist observations. These applications align with emerging priorities in AI-assisted adaptive therapy, which advocate real-time feedback and clinician-guided interpretability (Atturu et al., 2025; Ganai et al., 2025). The robustness of transition network metrics and flow-based saliency maps supports future work in live-feedback systems, where transitions through high-betweenness states or spikes in salient motor configurations could flag meaningful therapeutic inflection points.

### 5.2 Limitations and future directions

Notwithstanding these contributions, several limitations warrant consideration. The analyses were conducted on a small subset of the MMASD dataset (Li et al., 2023), which may limit statistical generalizability, an issue pervasive in ASD machine-learning studies (Rakotomanana and Rouhafzay, 2025). This study did not incorporate modalities such as gaze, facial key points, audio, or clinical outcome prediction (e.g., ADOS scores), which may be important for fuller clinical interpretation. Future work should integrate these channels to enhance clinical anchoring and validate against standardized assessment tools. The dominance of skeletal features may also reflect dataset bias (e.g., seated play tasks) rather than universal modality importance; future research should evaluate gross-motor contexts and larger samples. Moreover, while the discovered primitives are interpretable, they remain unsupervised and not yet mapped to standardized behavioural labels such as those in ADOS or clinical observation scales. Incorporating therapist annotations and outcome measures will be essential to establish external validity. Gradient-based saliency, while informative, is correlational rather than causal (Simonyan et al., 2013); integrating perturbation-based or counterfactual interpretability techniques could strengthen causal attribution. Future studies should also explore multi-scale temporal architectures (e.g., transformers or diffusion models) to capture longer-term learning dynamics.

## 6 Conclusion

This study introduces a comprehensive, explainable multimodal framework for modelling learning dynamics in autism-therapy interactions. By integrating skeletal pose, motion intensity, and temporal embeddings within an interpretable unsupervised architecture, we demonstrate that therapeutic behaviour unfolds through structured, low-dimensional trajectories rather than random motion. The framework quantifies adaptation, stability, and coordination, core dimensions of learning, through latent trajectories and network transitions. Critically, the alignment of model-derived features with clinical constructs (e.g., imitation, synchrony, engagement) bridges the gap between computational modelling and behavioural analysis in therapeutic contexts. While not diagnostic, the study introduces a process-oriented framework for understanding behavioural adaptation during intervention, representing a proof-of-concept toward transparent, data-driven personalization of therapy. Future work may align these trajectories with standardized clinical metrics such as ADOS scores or therapist-coded observations to support external validation. The framework’s ability to recover structured transition graphs, reproducible saliency profiles, and stable latent trajectories underscores its suitability for deployment in longitudinal, adaptive therapeutic systems.

## Data Availability

Publicly available datasets were analyzed in this study. This data can be found at: https://github.com/Li-Jicheng/MMASD-A-Multimodal-Dataset-for-Autism-Intervention-Analysis.git.

## Appendix

### Appendix A: Inference pipeline overview

1. Preprocessing

◦ Input: GIF/MP4 therapy videos
◦ Frame rate: 10–15 fps
◦ Windowing: 8-frame windows, stride = 4

2. Feature extraction

◦ Pose: 2D keypoints were extracted using MediaPipe BlazePose (33 joints) for all analysis. OpenPose BODY_25 (25 joints) was used only for visualization (e.g., Figure 1D), not for training or inference.
◦ Flow: Dense Farnebäck (OpenCV 4.10.0)
◦ Normalization: Z-score for flow; bbox-based for pose

3. Embedding

◦ Model: Temporal autoencoder (Gaussian noise: μ = 0, σ = 0.05 during training)
◦ Output: 128-dim latent vector per window

4. Primitive assignment

◦ Clustering: K-means (k = 6, seed = 42)
◦ Label: Closest centroid per window

5. Transition modeling

◦ Graph: Directed transitions per session
◦ Metrics: PageRank, betweenness, entropy (NetworkX 3.6.1)

6. Saliency analysis

◦ Method: Gradient × input
◦ Normalization: Z-score per joint
◦ Robustness: 5 random seeds, CV folds

## References

[ref1] Abdollahinejad Y. Kabir M. F. (2024). Enhancing Early Diagnosis of Autism Spectrum Disorder Using Multimodal Data and Explainable AI Models. Washington, DC, USA: IEEE.

[ref9001] Adebayo J. Gilmer J. Muelly M. Goodfellow I. Hardt M. Kim B. (2018). Sanity checks for saliency maps. Advances in neural information processing systems. 31., 34289458

[ref2] Ahmadi M. Kazemi K. Kuc K. Cybulska-Klosowicz A. Helfroush M. S. Aarabi A. (2021). Resting state dynamic functional connectivity in children with attention deficit/hyperactivity disorder. J. Neural Eng. 18:0460d1. doi: 10.1088/1741-2552/ac16b3, 34289458

[ref3] Ahmed Z. A. T. Albalawi E. Aldhyani T. H. H. Jadhav M. E. Janrao P. Obeidat M. R. M. (2023). Applying eye tracking with deep learning techniques for early-stage detection of autism spectrum disorders. Data 8:168. doi: 10.3390/data8110168

[ref4] Al-Beltagi M. (2021). Autism medical comorbidities. World J. Clin. Pediatr. 10, 15–28. doi: 10.5409/wjcp.v10.i3.15, 33972922 PMC8085719

[ref5] Ali S. Baloch Z. Narejo S. Shaikh A. M. (2025). A review of developments in generative AI, machine learning, and neuroimaging for the diagnosis of autism. VFAST Trans. Softw. Eng. 13, 68–82. doi: 10.21015/vtse.v13i2.2089

[ref6] Anzulewicz A. Sobota K. Delafield-Butt J. T. (2016). Toward the autism motor signature: gesture patterns during smart tablet gameplay identify children with autism. Sci. Rep. 6:31107. doi: 10.1038/srep31107, 27553971 PMC4995518

[ref7] Arias F. Nunez M. Z. Guerra-Adames A. Tejedor-Flores N. Vargas-Lombardo M. (2022). Sentiment analysis of public social media as a tool for health-related topics. IEEE Access 10, 74850–74872. doi: 10.1109/access.2022.3187406

[ref8] Arnab A. Dehghani M. Heigold G. Sun C. Lučić M. Schmid C. (2021). Vivit: A video vision transformer. New Jersey, USA: IEEE/CVF (IEEE Computer Society and the Computer Vision Foundation).

[ref9] Atturu H. Naraganti S. Rao B. R. (2025). Effectiveness of artificial intelligence–based platform in administering therapies for children with autism Spectrum disorder: 12-month observational study. JMIR Neurotechnol. 4:e70589. doi: 10.2196/70589, 41341424 PMC12671327

[ref10] Bahdanau D. Cho K. Bengio Y. (2014). Neural machine translation by jointly learning to align and translate. arXiv. doi: 10.48550/arXiv.1409.0473

[ref11] Bai D. Yip B. H. K. Windham G. C. Sourander A. Francis R. Yoffe R. . (2019). Association of genetic and environmental factors with autism in a 5-country cohort. JAMA Psychiatry 76, 1035–1043. doi: 10.1001/jamapsychiatry.2019.1411 31314057 PMC6646998

[ref12] Barbaro J. Dissanayake C. (2009). Autism spectrum disorders in infancy and toddlerhood: a review of the evidence on early signs, early identification tools, and early diagnosis. J. Dev. Behav. Pediatr. 30, 447–459. doi: 10.1097/DBP.0b013e3181ba0f9f, 19823139

[ref13] Bazarevsky V. Grishchenko I. Raveendran K. Zhu T. Zhang F. Grundmann M. (2020). Blazepose: on-device real-time body pose tracking. arXiv. doi: 10.48550/arXiv.2006.10204

[ref14] Bellosta P. Soldano A. (2019). Dissecting the genetics of autism spectrum disorders: a Drosophila perspective. Front. Physiol. 10:987. doi: 10.3389/fphys.2019.00987, 31481894 PMC6709880

[ref15] Bengio Y. Courville A. Vincent P. (2013). Representation learning: a review and new perspectives. IEEE Trans. Pattern Anal. Mach. Intell. 35, 1798–1828. doi: 10.1109/TPAMI.2013.50, 23787338

[ref16] Bhat A. N. Landa R. J. Galloway J. C. (2011). Current perspectives on motor functioning in infants, children, and adults with autism spectrum disorders. Phys. Ther. 91, 1116–1129. doi: 10.2522/ptj.20100294, 21546566

[ref17] Bölte S. Girdler S. Marschik P. B. (2019). The contribution of environmental exposure to the etiology of autism spectrum disorder. Cell. Mol. Life Sci. 76, 1275–1297. doi: 10.1007/s00018-018-2988-4, 30570672 PMC6420889

[ref18] Bottema-Beutel K. Kim S. Y. Crowley S. (2019). A systematic review and meta-regression analysis of social functioning correlates in autism and typical development. Autism Res. 12, 152–175. doi: 10.1002/aur.2055, 30575308

[ref19] Bottema-Beutel K. LaPoint S. C. Kim S. Y. Mohiuddin S. Yu Q. McKinnon R. (2023). An evaluation of intervention research for transition-age autistic youth. Autism 27, 890–904. doi: 10.1177/13623613221128761, 36189778

[ref20] Brin S. Page L. (1998). The anatomy of a large-scale hypertextual web search engine. Comput. Netw. ISDN Syst. 30, 107–117. doi: 10.1016/s0169-7552(98)00110-x

[ref21] Charalambous E. Efstratopoulou M. (2025). “Artificial intelligence approaches to autism Spectrum disorder screening and diagnosis,” in AI in Mental Health: Innovations, Challenges, and Collaborative Pathways, (IGI Global Scientific Publishing), 41–70.

[ref22] Cheroni C. Caporale N. Testa G. (2020). Autism spectrum disorder at the crossroad between genes and environment: contributions, convergences, and interactions in ASD developmental pathophysiology. Mol. Autism. 11:69. doi: 10.1186/s13229-020-00370-1, 32912338 PMC7488083

[ref23] Chong E. Chanda K. Ye Z. Southerland A. Ruiz N. Jones R. M. . (2017). Detecting gaze towards eyes in natural social interactions and its use in child assessment. arXiv 1, 1–20. doi: 10.1145/3131902

[ref24] Chung K.-M. Chung E. Lee H. (2024). Behavioral interventions for autism spectrum disorder: a brief review and guidelines with a specific focus on applied behavior analysis. J. Korean Acad. Child Adolesc. Psychiatry 35, 29–38. doi: 10.5765/jkacap.230019, 38204739 PMC10774556

[ref25] Cruz Puerto M. Sandín Vázquez M. (2024). Understanding heterogeneity within autism spectrum disorder: a scoping review. Adv. Autism 10, 314–322. doi: 10.1108/aia-12-2023-0072

[ref26] Daniel E. Gulati A. Saxena S. Urgun D. A. Bista B. (2025). GM-VGG-net: a gray matter-based deep learning network for autism classification. Diagnostics 15:1425. doi: 10.3390/diagnostics15111425, 40506998 PMC12154016

[ref27] Dawson G. Campbell K. Hashemi J. Lippmann S. J. Smith V. Carpenter K. . (2018). Atypical postural control can be detected via computer vision analysis in toddlers with autism spectrum disorder. Sci. Rep. 8:17008. doi: 10.1038/s41598-018-35215-8, 30451886 PMC6242931

[ref28] Dcouto S. S. Pradeepkandhasamy J. (2024). Multimodal deep learning in early autism detection—recent advances and challenges. Eng. Proc. 59:205. doi: 10.3390/engproc2023059205

[ref29] De Belen R. A. J. Bednarz T. Sowmya A. Del Favero D. (2020). Computer vision in autism spectrum disorder research: a systematic review of published studies from 2009 to 2019. Transl. Psychiatry 10:333. doi: 10.1038/s41398-020-01015-w, 32999273 PMC7528087

[ref30] Di Martino A. Yan C.-G. Li Q. Denio E. Castellanos F. X. Alaerts K. . (2014). The autism brain imaging data exchange: towards a large-scale evaluation of the intrinsic brain architecture in autism. Mol. Psychiatry 19, 659–667. doi: 10.1038/mp.2013.78, 23774715 PMC4162310

[ref31] Dickstein-Fischer L. Fischer G. S. (2014) Combining Psychological and Engineering Approaches to Utilizing social Robots with children with Autism. International Journal of Biological and Pharmaceutical Sciences Archive. Chicago, IL, USA: IEEE. 10.1109/EMBC.2014.6943710 25570078

[ref32] Dos Santos Melicio B. C. Karaköse K. Fodor Á. Xiang L. Varga V. Soorya L. . (2025). Multimodal framework for automatic behavior analysis of children with autism during ADOS-2. Cogn. Comput. 17, 1–17. doi: 10.1007/s12559-025-10481-7, 30311153

[ref33] Ecker C. Murphy D. (2014). Neuroimaging in autism—from basic science to translational research. Nat. Rev. Neurol. 10, 82–91. doi: 10.1038/nrneurol.2013.276, 24419683

[ref34] Egbuna I. K. Otoibhili P. A. Abdulkareem R. O. Ikechukwu F. Ogbozor P. E. O. Azih N. K. . (2025). Explainable Artificial Intelligence (XAI) in Diagnosing Neurodevelopmental Disorders: From Black Boxes to Clinical Transparency

[ref35] Eldin Rashed A. E. Bahgat W. M. Ahmed A. Ahmed Farrag T. Mansour Atwa A. E. (2025). Efficient machine learning models across multiple datasets for autism spectrum disorder diagnoses. Biomed. Signal Process. Control 100:106949. doi: 10.1016/j.bspc.2024.106949

[ref36] Esubalew T. Lahiri U. Swanson A. R. Crittendon J. A. Warren Z. E. Sarkar N. (2012). A step towards developing adaptive robot-mediated intervention architecture (ARIA) for children with autism. IEEE Trans. Neural Syst. Rehabil. Eng. 21, 289–299. doi: 10.1109/TNSRE.2012.2230188 23221831 PMC3860752

[ref37] Farnebäck G. (2003). Two-frame Motion Estimation based on Polynomial Expansion. Springer.

[ref38] Farooq M. S. Tehseen R. Sabir M. Atal Z. (2023). Detection of autism spectrum disorder (ASD) in children and adults using machine learning. Sci. Rep. 13:9605. doi: 10.1038/s41598-023-35910-1, 37311766 PMC10264444

[ref39] Freeman L. C. (1977). A set of measures of centrality based on betweenness. Sociometry 40, 35–41. doi: 10.2307/3033543

[ref40] Ganai U. J. Ratne A. Bhushan B. Venkatesh K. S. (2025). Early detection of autism spectrum disorder: gait deviations and machine learning. Sci. Rep. 15:873. doi: 10.1038/s41598-025-85348-w, 39757284 PMC11701103

[ref41] Gardner L. M. Campbell J. M. Keisling B. Murphy L. (2018). Correlates of DSM-5 autism spectrum disorder levels of support ratings in a clinical sample. J. Autism Dev. Disord. 48, 3513–3523. doi: 10.1007/s10803-018-3620-z, 29845346

[ref42] Gardner-Hoag J. Novack M. Parlett-Pelleriti C. Stevens E. Dixon D. Linstead E. (2021). Unsupervised machine learning for identifying challenging behavior profiles to explore cluster-based treatment efficacy in children with autism spectrum disorder: retrospective data analysis study. JMIR Med. Inform. 9:e27793. doi: 10.2196/27793, 34076577 PMC8209527

[ref43] Giansanti D. (2023). An umbrella review of the fusion of fMRI and AI in autism. Diagnostics 13:3552. doi: 10.3390/diagnostics13233552, 38066793 PMC10706112

[ref44] Gillberg C. (2021). The Essence of Autism and other Neurodevelopmental Conditions: Rethinking co-Morbidities, Assessment, and Intervention. London: Philadelphia.

[ref45] Gkintoni E. Panagioti M. Vassilopoulos S. P. Nikolaou G. Boutsinas B. Vantarakis A. (2025). Leveraging AI-driven neuroimaging biomarkers for early detection and social function prediction in autism Spectrum disorders: a systematic review. MDPI. 10.3390/healthcare13151776 PMC12346713 40805809

[ref46] Guo Z. Chheang V. Li J. Barner K. E. Bhat A. Barmaki R. L. (2023). Social visual Behavior Analytics for Autism Therapy of children based on Automated Mutual gaze Detection. New York, NY, USA: ACM.

[ref47] Harrold N. Tan C. T. Rosser D. Leong T. W. (2014). “CopyMe: a portable real-time feedback expression recognition game for children,” in CHI'14 Extended Abstracts on human Factors in Computing Systems, (), 1195–1200. doi: 10.1145/2559206.2581279

[ref9002] Hashemi J. Spina T. V. Tepper M. Esler A. Morellas B. Papanikolopoulos N. . (2012). Computer vision tools for the non-invasive assessment of autism-related behavioral markers. arXiv preprint arXiv:1210.7014., 40031756

[ref48] Hassan I. Nahid N. Islam M. Hossain S. Schuller B. Ahad M. A. R. (2025). Automated autism assessment with multimodal data and ensemble learning: a scalable and consistent robot-enhanced therapy framework. IEEE Trans. Neural Syst. Rehabil. Eng. 33, 1191–1201. doi: 10.1109/TNSRE.2025.3546519, 40031756

[ref49] Hinton G. E. Salakhutdinov R. R. (2006). Reducing the dimensionality of data with neural networks. Science 313, 504–507. doi: 10.1126/science.1127647, 16873662

[ref50] Hofmann H. A. Beery A. K. Blumstein D. T. Couzin I. D. Earley R. L. Hayes L. D. . (2014). An evolutionary framework for studying mechanisms of social behavior. Trends Ecol. Evol. 29, 581–589. doi: 10.1016/j.tree.2014.07.008, 25154769

[ref51] Hossain M. D. Ahmed H. U. Jalal Uddin M. M. Chowdhury W. A. Iqbal M. S. Kabir R. I. . (2017). Autism spectrum disorders (ASD) in South Asia: a systematic review. BMC Psychiatry 17:281. doi: 10.1186/s12888-017-1440-x, 28826398 PMC5563911

[ref52] Huber P. J. (1992). “Robust estimation of a location parameter,” in Breakthroughs in Statistics: Methodology and Distribution, (New York: Springer), 492–518.

[ref53] Hus Y. Segal O. (2021). Challenges surrounding the diagnosis of autism in children. Neuropsychiatr. Dis. Treat. 17, 3509–3529. doi: 10.2147/NDT.S282569, 34898983 PMC8654688

[ref54] Jolliffe I. (2011). “Principal component analysis,” in International Encyclopedia of Statistical Science, (Berlin: Springer), 1094–1096.

[ref55] Khan K. Katarya R. (2025). MCBERT: a multi-modal framework for the diagnosis of autism spectrum disorder. Biol. Psychol. 194:108976. doi: 10.1016/j.biopsycho.2024.108976, 39722324

[ref56] Khosla M. Jamison K. Kuceyeski A. Sabuncu M. R. (2019a). Ensemble learning with 3D convolutional neural networks for functional connectome-based prediction. NeuroImage 199, 651–662. doi: 10.1016/j.neuroimage.2019.06.012, 31220576 PMC6777738

[ref57] Khosla M. Jamison K. Ngo G. H. Kuceyeski A. Sabuncu M. R. (2019b). Machine learning in resting-state fMRI analysis. Magn. Reson. Imaging 64, 101–121. doi: 10.1016/j.mri.2019.05.031, 31173849 PMC6875692

[ref58] Kindregan D. Gallagher L. Gormley J. (2015). Gait deviations in children with autism spectrum disorders: a review. Autism Res. Treat. 2015:741480. doi: 10.1155/2015/741480, 25922766 PMC4398922

[ref59] King C. Merrick H. Le Couteur A. (2020). How should we support young people with ASD and mental health problems as they navigate the transition to adult life including access to adult healthcare services. Epidemiol. Psychiatr. Sci. 29:e90. doi: 10.1017/S2045796019000830, 31915102 PMC7214707

[ref60] Kojovic N. Natraj S. Mohanty S. P. Maillart T. Schaer M. (2021). Using 2D video-based pose estimation for automated prediction of autism spectrum disorders in young children. Sci. Rep. 11:15069. doi: 10.1038/s41598-021-94378-z, 34301963 PMC8302646

[ref61] Kullback S. Leibler R. A. (1951). On information and sufficiency. Ann. Math. Stat. 22, 79–86. doi: 10.1214/aoms/1177729694

[ref62] Kwon J. Kotani H. (2025). Quantifying body motion synchrony in autism Spectrum disorder using a phase difference detection algorithm: toward a novel behavioral biomarker. Diagnostics 15:1268. doi: 10.3390/diagnostics15101268, 40428261 PMC12110654

[ref63] Lai M.-C. Lombardo M. V. Auyeung B. Chakrabarti B. Baron-Cohen S. (2015). Sex/gender differences and autism: setting the scene for future research. J. Am. Acad. Child Adolesc. Psychiatry 54, 11–24. doi: 10.1016/j.jaac.2014.10.003, 25524786 PMC4284309

[ref64] Landa R. J. Holman K. C. Garrett-Mayer E. (2007). Social and communication development in toddlers with early and later diagnosis of autism spectrum disorders. Arch. Gen. Psychiatry 64, 853–864. doi: 10.1001/archpsyc.64.7.853, 17606819

[ref65] LeCun Y. Bengio Y. Hinton G. (2015). Deep learning. Nature 521, 436–444. doi: 10.1038/nature14539, 26017442

[ref66] Lee K. Lambert H. Wittich W. Kehayia E. Park M. (2016). The use of movement-based interventions with children diagnosed with autism for psychosocial outcomes—a scoping review. Res. Autism Spectr. Disord. 24, 52–67. doi: 10.1016/j.rasd.2015.12.011

[ref67] Leekam S. Tandos J. McConachie H. Meins E. Parkinson K. Wright C. . (2007). Repetitive behaviours in typically developing 2-year-olds. J. Child Psychol. Psychiatry 48, 1131–1138. doi: 10.1111/j.1469-7610.2007.01778.x, 17995489

[ref68] Leo M. Sharma S. Maddulety K. (2019). Machine learning in banking risk management: a literature review. Risks 7:29. doi: 10.3390/risks7010029

[ref69] Li J. Chheang V. Kullu P. Brignac E. Guo Z. Bhat A. . (2023). Mmasd: A Multimodal Dataset for Autism Intervention Analysis. doi: 10.48550/arXiv.2306.08243

[ref70] Liu M. Liu H. Hu Q. Ren B. Yuan J. Lin J. . (2025). 3d skeleton-based action recognition: a review. arXiv. doi: 10.48550/arXiv.2506.00915

[ref71] Lloyd S. (1982). Least squares quantization in PCM. IEEE Trans. Inf. Theory 28, 129–137. doi: 10.1109/tit.1982.1056489

[ref72] Lord C. Risi S. Lambrecht L. Cook E. H. Jr. Leventhal B. L. DiLavore P. C. . (2000). The autism diagnostic observation schedule—generic: a standard measure of social and communication deficits associated with the spectrum of autism. J. Autism Dev. Disord. 30, 205–223. doi: 10.1023/A:1005592401947, 11055457

[ref73] Maaten L. V. D. Hinton G. (2008). Visualizing data using t-SNE. J. Mach. Learn. Res. 9, 2579–2605.

[ref74] Maenner M. J. (2023). Prevalence and characteristics of autism spectrum disorder among children aged 8 years—autism and developmental disabilities monitoring network, 11 sites, United States, 2020 Morbidity and mortality weekly report. Surveill. Summ. 72, 1–14. doi: 10.15585/mmwr.ss7202a1, 36952288 PMC10042614

[ref75] Magrini M. Carboni A. Salvetti O. Curzio O. (2015). An Auditory Feedback based system for Treating Autism spectrum Disorder. doi: 10.1145/2838944.2838952

[ref76] Malik W. Fahiem M. A. Farhat T. Alghazo R. Mahmood A. Alhajlah M. (2025). An explainable deep learning framework for multimodal autism diagnosis using XAI GAMI-net and Hypernetworks. Diagnostics 15:2232. doi: 10.3390/diagnostics15172232, 40941719 PMC12427627

[ref77] Martin K. B. Hammal Z. Ren G. Cohn J. F. Cassell J. Ogihara M. . (2018). Objective measurement of head movement differences in children with and without autism spectrum disorder. Mol. Autism. 9:14. doi: 10.1186/s13229-018-0198-4, 29492241 PMC5828311

[ref78] Mazzei D. Greco A. Lazzeri N. Zaraki A. Lanata A. Igliozzi R. . (2012) Robotic social Therapy on children with Autism: Preliminary Evaluation through multi-Parametric Analysis. Amsterdam, Netherlands: IEEE.

[ref79] McCleery J. P. Elliott N. A. Sampanis D. S. Stefanidou C. A. (2013). Motor development and motor resonance difficulties in autism: relevance to early intervention for language and communication skills. Front. Integr. Neurosci. 7:30. doi: 10.3389/fnint.2013.00030, 23630476 PMC3634796

[ref80] McQueen J. B. (1967) Some Methods of Classification and Analysis of Multivariate Observations. Berkeley, California, USA: University of California Press.

[ref81] Miao K. Hounye A. H. Su L. Pan Q. Wang J. Hou M. . (2024). Exploring explainable machine learning and Shapley additive exPlanations (SHAP) technique to uncover key factors of HNSC cancer: an analysis of the best practices. Biomed. Signal Process. Control 89:105752. doi: 10.1016/j.bspc.2023.105752

[ref82] Ohmoto Y. Terada K. Shimizu H. Kawahara H. Iwanaga R. Kumazaki H. (2024). Machine learning’s effectiveness in evaluating movement in one-legged standing test for predicting high autistic trait. Front. Psych. 15:1464285. doi: 10.3389/fpsyt.2024.1464285, 39483737 PMC11524919

[ref83] Pagnozzi A. M. Conti E. Calderoni S. Fripp J. Rose S. E. (2018). A systematic review of structural MRI biomarkers in autism spectrum disorder: a machine learning perspective. Int. J. Dev. Neurosci. 71, 68–82. doi: 10.1016/j.ijdevneu.2018.08.010, 30172895

[ref84] Parlett-Pelleriti C. M. Stevens E. Dixon D. Linstead E. J. (2023). Applications of unsupervised machine learning in autism spectrum disorder research: a review. Rev. J. Autism Dev. Disord. 10, 406–421. doi: 10.1007/s40489-021-00299-y

[ref85] Piana S. Malagoli C. Usai M. C. Camurri A. (2019). Effects of computerized emotional training on children with high functioning autism. IEEE Trans. Affect. Comput. 12, 1045–1054. doi: 10.1109/taffc.2019.2916023

[ref86] Qian R. Meng T. Gong B. Yang M.-H. Wang H. Belongie S. . (2021). Spatiotemporal Contrastive video Representation Learning IEEE Computer Society.

[ref87] Rakotomanana H. Rouhafzay G. (2025). A scoping review of AI-based approaches for detecting autism traits using voice and behavioral data. Bioengineering 12:1136. doi: 10.3390/bioengineering12111136, 41301092 PMC12649475

[ref88] Ringland K. E. Zalapa R. Neal M. Escobedo L. Tentori M. E. Hayes G. R. (2014). “SensoryPaint: a natural user interface supporting sensory integration in children with neurodevelopmental disorders,” in CHI'14 Extended Abstracts on Human Factors in Computing Systems, (New York, NY, USA: ACM.) 1681–1686.

[ref89] Robain F. Franchini M. Kojovic N. Wood de Wilde H. Schaer M. (2020). Predictors of treatment outcome in preschoolers with autism spectrum disorder: an observational study in the greater Geneva area, Switzerland. J. Autism Dev. Disord. 50, 3815–3830. doi: 10.1007/s10803-020-04430-6, 32166526

[ref90] Rodgers H. R. McCluney J. (2021). Prevalence of Autism (Including Asperger Syndrome) in School Age Children in Northern Ireland: Annual Report 2021. Department of Health (Northern Ireland).

[ref91] Roth A. Ullman J. Wu Z. S. (2016). Watch and learn: Optimizing from Revealed Preferences Feedback. New York, NY, United States: ACM.

[ref92] Saha P. Tapotee M. I. Ahad M. A. R. (2021). Task Detection of ASD children by Analyzing Robotic Enhanced and Standard human Therapy. Tokyo, Japan: IEEE.

[ref93] Sandbank M. Bottema-Beutel K. Crowley S. Cassidy M. Dunham K. Feldman J. I. . (2020). Project AIM: autism intervention meta-analysis for studies of young children. Psychol. Bull. 146, 1–29. doi: 10.1037/bul0000215, 31763860 PMC8783568

[ref94] Scherzer A. L. Chhagan M. Kauchali S. Susser E. (2012). Global perspective on early diagnosis and intervention for children with developmental delays and disabilities. Dev. Med. Child Neurol. 54, 1079–1084. doi: 10.1111/j.1469-8749.2012.04348.x, 22803576 PMC3840420

[ref95] Schmidhuber J. (2015). Deep learning. Scholarpedia 10:32832. doi: 10.4249/scholarpedia.32832, 25462637

[ref96] Sealey L. A. Hughes B. W. Sriskanda A. N. Guest J. R. Gibson A. D. Johnson-Williams L. . (2016). Environmental factors in the development of autism spectrum disorders. Environ. Int. 88, 288–298. doi: 10.1016/j.envint.2015.12.021, 26826339

[ref97] Sengupta K. Lobo L. Krishnamurthy V. (2017). Educational and behavioral interventions in management of autism spectrum disorder. Indian J. Pediatrics 84, 61–67. doi: 10.1007/s12098-015-1967-0, 26661442

[ref98] Shamhan A. N. M. Qaraqe M. Al-Thani D. (2025). Advancements in automated assessment and diagnosis of autism spectrum disorder through multi-modality sensing technologies: survey of the last decade. IEEE Trans. Cogn. Dev. Syst. 17, 727–745. doi: 10.1109/tcds.2025.3574145

[ref99] Shamseddine H. Otoum S. Mourad A. (2022). On the Feasibility of Federated Learning for Neurodevelopmental Disorders: Asd Detection use-case. Rio de Janeiro, Brazil: IEEE.

[ref100] Shaw C. A. Sheth S. Li D. Tomljenovic L. (2014). Etiology of autism spectrum disorders: genes, environment, or both. OA Autism 2:11.

[ref101] Simonyan K. Vedaldi A. Zisserman A. (2013). Deep inside convolutional networks: visualising image classification models and saliency maps. arXiv. doi: 10.48550/arXiv.1312.6034

[ref102] Song T. Ren Z. Zhang J. Wang M. (2024). Multi-view and multimodal graph convolutional neural network for autism spectrum disorder diagnosis. Mathematics 12:1648. doi: 10.3390/math12111648

[ref103] Steinbrenner J. R. Hume K. Odom S. L. Morin K. L. Nowell S. W. Tomaszewski B. . (2020). Evidence-based Practices for children, Youth, and young Adults with Autism. Chapel Hill, NC, United States: FPG child development institute.

[ref104] Stevens E. Dixon D. R. Novack M. N. Granpeesheh D. Smith T. Linstead E. (2019). Identification and analysis of behavioral phenotypes in autism spectrum disorder via unsupervised machine learning. Int. J. Med. Inform. 129, 29–36. doi: 10.1016/j.ijmedinf.2019.05.006, 31445269

[ref105] Stewart L. A. Lee L.-C. (2017). Screening for autism spectrum disorder in low-and middle-income countries: a systematic review. Autism 21, 527–539. doi: 10.1177/1362361316677025, 28183195

[ref106] Taylor L. J. Eapen V. Maybery M. Midford S. Paynter J. Quarmby L. . (2017). Brief report: an exploratory study of the diagnostic reliability for autism spectrum disorder. J. Autism Dev. Disord. 47, 1551–1558. doi: 10.1007/s10803-017-3054-z, 28233080

[ref107] Teitelbaum P. Teitelbaum O. Nye J. Fryman J. Maurer R. G. (1998). Movement analysis in infancy may be useful for early diagnosis of autism. Proc. Natl. Acad. Sci. 95, 13982–13987. doi: 10.1073/pnas.95.23.13982, 9811912 PMC25000

[ref108] Toaiari A. (2025) Deep Learning for Human Behaviour Understanding: A Comprehensive Study of Trajectory, Pose, and Gaze in Social and Human-Robot Interaction Scenarios. Verona, Italy: University of Verona.

[ref109] Torres E. B. Travers B. G. Delafield-Butt J. T. Srinivasan A. (2025). Autism: The Movement (Sensing) Perspective a Decade Later, vol. 19 Frontiers Media SA, Switzerland 1634265. 10.3389/fnint.2025.1634265 PMC12222190 40613074

[ref110] Torres E. B. Yanovich P. Metaxas D. N. (2013). Give spontaneity and self-discovery a chance in ASD: spontaneous peripheral limb variability as a proxy to evoke centrally driven intentional acts. Front. Integr. Neurosci. 7:46. doi: 10.3389/fnint.2013.00046, 23898243 PMC3721359

[ref111] Tsai M.-F. Chen C.-H. (2021). Spatial temporal variation graph convolutional networks (STV-GCN) for skeleton-based emotional action recognition. IEEE Access 9, 13870–13877. doi: 10.1109/access.2021.3052246

[ref112] Twala B. Molloy E. (2023). On effectively predicting autism spectrum disorder therapy using an ensemble of classifiers. Sci. Rep. 13:19957. doi: 10.1038/s41598-023-46379-3, 37968315 PMC10651853

[ref113] Usmani S. Siddiqui Y. A. Hamza M. Shaikh M. T. Hasan Q. (2024). Exploring the Landscape of Multimodal Generative Adversarial Networks: A Comprehensive Survey and Analysis. Academic Press.

[ref114] Vaswani A. Shazeer N. Parmar N. Uszkoreit J. Jones L. Gomez A. N. . (2017). “Attention is all you need,” in Advances in Neural Information Processing Systems, (Long Beach California USA: Curran Associates, Inc.), 30.

[ref115] Vidya S. Gupta K. Aly A. Wills A. Ifeachor E. Shankar R. (2024). Explainable AI for autism diagnosis: identifying critical brain regions using fMRI data. arXiv. doi: 10.48550/arXiv.2409.15374 PMC12573454 41181843

[ref116] Visser J. C. Rommelse N. N. Greven C. U. Buitelaar J. K. (2016). Autism spectrum disorder and attention-deficit/hyperactivity disorder in early childhood: a review of unique and shared characteristics and developmental antecedents. Neurosci. Biobehav. Rev. 65, 229–263. doi: 10.1016/j.neubiorev.2016.03.019, 27026637

[ref117] Volkmar F. R. McPartland J. C. (2014). From Kanner to DSM-5: autism as an evolving diagnostic concept. Annu. Rev. Clin. Psychol. 10, 193–212. doi: 10.1146/annurev-clinpsy-032813-153710, 24329180

[ref118] Wang X. Gao H. Zhang Y. Jiang Y. Yu J. (2025). Visual human behavior sensing and understanding for autism spectrum disorder treatment: a review. Sens. Mater. 37:1007. doi: 10.18494/sam4468

[ref119] Wang M. Yang N. (2022). OTA-NN: Observational Therapy-Assistance Neural Network for Enhancing Autism Intervention Quality. Las Vegas, NV, USA: IEEE.

[ref120] Wei Q. Dong W. Yu D. Wang K. Yang T. Xiao Y. . (2024). Early identification of autism spectrum disorder based on machine learning with eye-tracking data. J. Affect. Disord. 358, 326–334. doi: 10.1016/j.jad.2024.04.049, 38615846

[ref121] Weng S. F. Reps J. Kai J. Garibaldi J. M. Qureshi N. (2017). Can machine-learning improve cardiovascular risk prediction using routine clinical data? PLoS One 12:e0174944. doi: 10.1371/journal.pone.0174944, 28376093 PMC5380334

[ref122] White S. W. Abbott L. Wieckowski A. T. Capriola-Hall N. N. Aly S. Youssef A. (2018). Feasibility of automated training for facial emotion expression and recognition in autism. Behav. Ther. 49, 881–888. doi: 10.1016/j.beth.2017.12.010, 30316487

[ref123] Wiggins L. D. Baio J. O. N. Rice C. (2006). Examination of the time between first evaluation and first autism spectrum diagnosis in a population-based sample. J. Dev. Behav. Pediatr. 27, S79–S87. doi: 10.1097/00004703-200604002-00005, 16685189

[ref124] Yan S. Xiong Y. Lin D. (2018). Spatial temporal graph Convolutional Networks for skeleton-based action Recognition, vol. 32. New Orleans, Louisiana, USA: AAAI Press.

[ref125] Yang X. Li S. Niu S. Yue X. (2026). Graph network learning for human skeleton modeling: a survey. Artif. Intell. Rev. 59:31. doi: 10.1007/s10462-025-11442-0, 30311153

[ref126] Yang Z. Zhang Y. Ning J. Wang X. Wu Z. (2025). Early diagnosis of autism: a review of video-based motion analysis and deep learning techniques. IEEE Access 13, 2903–2928. doi: 10.1109/access.2024.3523872

[ref127] Yoo H. (2016). Early detection and intervention of autism spectrum disorder. Hanyang Med. Rev. 36, 4–10. doi: 10.7599/hmr.2016.36.1.4

[ref128] Zampella C. J. Wang L. A. L. Haley M. Hutchinson A. G. de Marchena A. (2021). Motor skill differences in autism spectrum disorder: a clinically focused review. Curr. Psychiatry Rep. 23:64. doi: 10.1007/s11920-021-01280-6, 34387753

[ref129] Zhao J. Huang J. Zhi D. Yan W. Ma X. Yang X. . (2020). Functional network connectivity (FNC)-based generative adversarial network (GAN) and its applications in classification of mental disorders. J. Neurosci. Methods 341:108756. doi: 10.1016/j.jneumeth.2020.108756, 32380227 PMC7367699

[ref130] Zhou B. Andonian A. Oliva A. Torralba A. (2018). Temporal Relational Reasoning in Videos. Springer Nature.

